# Familial Reclassification Within Order *Lysobacterales* and Proposal of Four Novel Species

**DOI:** 10.3390/microorganisms13061212

**Published:** 2025-05-26

**Authors:** Tengfei Ma, Haijiao Liu, Yafei Chen, Juan Liu, Chungen Piao, Han Xue, Risheng Xu, Yong Li

**Affiliations:** 1 Peanut Research Institute, Henan Academy of Agricultural Sciences, Zhengzhou 450002, Chinahnpp2010@163.com (Y.C.);; 2 Key Laboratory of Biodiversity Conservation of National Forestry and Grassland Administration, Ecology and Nature Conservation Institute, Chinese Academy of Forestry, Beijing 100091, China

**Keywords:** *Lysobacterales*, phylogenomic, reannotation taxonomy, bacterial core genes

## Abstract

The order *Lysobacterales* consists of three families (*Rhodanobacteraceae*, *Lysobacteraceae* and *Marinicellaceae*), many members of which are important pathogenic and beneficial bacteria. Previous classifications of members within order *Lysobacterales* have relied heavily on 16S rRNA gene sequences, leading to taxonomic ambiguities at the familial level. With the advancement of sequencing technologies, an increasing number of whole-genome sequences have been available, providing an opportunity to revisit the taxonomy of families in *Lysobacterales*. In this study, we revisited the taxonomy of *Lysobacterales* by focusing on family-level reclassification based on phylogenomic frameworks. A total of 218 genome sequences, including 217 strains from *Lysobacterales* and 1 from *Nevskiales* (used as an outgroup), were collected for phylogenetic analysis. Phylogenetic relationships were inferred based on UBCG (up-to-date bacterial core gene) approach using 92 core genes and a concatenated protein phylogeney based on 227 single-copy orthologous proteins. Additionally, genomic similarity metrics, including average nucleotide identity (ANI), digital DNA–DNA hybridization (dDDH), average amino acid identity (AAI) and core-proteome average amino acid identity (cpAAI), were employed to assess the taxonomy of order *Lysobacterales*. Our results support the proposal of one novel family and the reassignment of six genera across different families within *Lysobacterales*, emphasizing the need for a refined family-level taxonomy. In addition, four novel species belonging to the family *Lysobacteraceae* were also confirmed. This study provides an updated familial framework for *Lysobacterales*, laying a robust foundation for future detailed taxonomic revisions at the genus and species levels.

## 1. Introduction

The order *Lysobacterales* (formerly *Xanthomonadales*) taxonomically belongs to the class *Gammaproteobacteria* [1] and currently comprises 3 families, 31 genera and 267 species with validly published names. The family *Lysobacteraceae* includes 18 genera and 200 species; *Rhodanobacteraceae* contains 15 genera and 74 species; and *Marinicellaceae*, proposed recently, consists of 1 genus and 7 species with validly published names (LSPN database). Historically, the order *Lysobacterales* included five families: *Nevskiaceae*, *Xanthomonadaceae*, *Algiphilaceae*, *Solimonadaceae* and *Sinobacteraceae*. In 2015, *Nevskiaceae*, *Algiphilaceae*, *Solimonadaceae* and *Sinobacteraceae* were transferred to a newly established order, *Nevskiales*, while the remaining members were reclassified into two families, *Lysobacteraceae* and *Rhodanobacteraceae*, based on phylogenetic analyses of 25 conserved proteins and conserved signature indels [2]. Recently, two additional families (*Ahniellaceae*, with not yet validly published names, and *Marinicellaceae*, with validly published names) were proposed according to the Genome Taxonomy Database (GTDB) [3]. However, *Marinicellaceae* should belong to a different order outside *Lysobacterales*. Therefore, in the subsequent analyses of this study, we focused only on three families within *Lysobacterales*: *Ahniellaceae*, *Lysobacteraceae* and *Rhodanobacteraceae*. Moreover, due to the limited availability of genomic data, several genera within *Lysobacterales* have not been properly classified at the family level in previous studies, resulting in misclassifications.

Although there is no consensus on a standardized metric for genus- or higher-level classification, several phylogenomic indices (ANI, AAI, cpAAI) could be chosen as supplementary evidence to support the creation of new genera (or higher levels) [4,5]. Genome-based phylogeny has been regarded as a more effective approach for genus- or family-level classification [6], and it is recommended that at least 30 single-copy orthologous genes be used in phylogenomic analysis [7]. In this paper, phylogenetic analyses based on 16S rRNA gene, 92 core genes (UBCG) and the concatenated alignment of 227 single-copy orthologous proteins, combined with ANI, AAI and cpAAI metrics, were conducted to investigate the taxonomic relationships of families within *Lysobacterales*. Additionally, this study also describes the isolation, identification and description of four novel species. These reclassifications not only resolve previous taxonomic inconsistencies but also provide a clearer framework for understanding the ecological roles of *Lysobacterales* members, including their potential as plant pathogens or beneficial microbes in biocontrol applications.

## 2. Materials and Methods

### 2.1. Strain and Culture Conditions

Strain MHLX1A^T^ was isolated from leaf spot disease of *Quercus dentata* collected from Beijing, China (40°00′14″ N, 116°14′24″ E); strain BDR2-5^T^ was isolated from bark sample of *Quercus acutissima* collected from Anhui, China (31°43′15″ N, 117°0′45″ E); and strains XNQY3-4^T^ and Y-2-3-4F^T^ were isolated from bark canker of *Populus euramericana* collected from Qinghai, China (36°36′26″ N, 101°46′39″ E), and Ningxia, China (37°31 55″ N, 105°5′46″ E), respectively. Isolation followed previously established protocols [8]. In brief, samples were successively washed with 70% ethanol and 4% sodium hypochlorite to eliminate surface contaminants, then ground in a sterile mortar using a pestle. After incubating at 30 °C on TSA plates for two days, individual colonies were selected, transferred to a new plate and stored at −80 °C in 20% (*v*/*v*) glycerol.

### 2.2. Genomic Data

The genomes of the four novel strains were sequenced with Illumina NovaSeq PE150 (Illumina, San Diego, CA, USA). To ensure high-quality data, raw sequence reads were processed by filtering out low-quality reads, followed by de novo assembly using several bioinformatics tools, including SOAPdenovo (version 2.04) [9,10], SPAdes [11] and ABySS [12]. The assembled results were integrated with CISA [13], and gaps were filled using GapCloser (version 1.12). The whole genome sequences of the four novel strains were uploaded to the NCBI database (Table S1).

The genome sequences of reference type strains used in present study were obtained from NCBI database. These genomes were subjected to quality control procedures using CheckM [14], excluding those with contamination levels exceeding 5% or completeness below 95%. As a result, a total of 218 high-quality genome sequences, including the type strains of 217 *Lysobacterales* and 1 *Nevskiales*, were retained for further analysis, as listed in Table S1.

### 2.3. Phylogenetic Analyses

The full-length 16S rRNA gene sequences were extracted from the genome sequences via RNAmmer 1.2 [15] for phylogenetic analysis. All of the extracted full-length 16S rRNA gene sequences were aligned using CLUSTAL W, and the full-length 16S rRNA gene tree was constructed with MEGA X via maximum-parsimony, neighbor-joining and maximum-likelihood methods [16]. Tree reliability was assessed with 1000 bootstrap replicates.

Phylogenomic analysis, particularly through concatenated core gene trees, has become a standard approach for inferring evolutionary relationships due to its high-resolution capabilities [17]. Core genes from the species analyzed in this study were identified using UBCG [18], yielding 92 core genes via the command “java -jar UBCG.jar extract”. The phylogenomic tree was constructed via the command “java -jar UBCG.jar align”.

In addition, a concatenated protein phylogeny was also performed with OrthoFinder [19] and FastTree [20]. In brief, the whole genome protein sequences of 217 *Lysobacterales* type strains were first predicted by prodigal [21], then the single-copy orthologous proteins were extracted from the sequences by OrthoFinder with default parameters, and 227 single-copy orthologous proteins were obtained. Finaly a concatenated alignment of the 227 single-copy proteins was used to construct the phylogenetic tree with FastTree. The visualization and editing of all phylogenetic trees were completed with iTOL [22].

### 2.4. Genome-Based Metrics Analyses

Average nucleotide identity (ANI) values serve as a key metric for species classification and were calculated using pyani [23]. Along with ANI, the digital DNA–DNA hybridization (dDDH) values, another metric for species classification, were calculated with the GGDC (http://ggdc.dsmz.de, accessed on 16 July 2024). For higher taxonomic ranks, average amino acid identity (AAI) was determined by using CompareM [24]. In addition, we also calculated the core-proteome average amino acid identity (cpAAI) of 217 *Lysobacterales* type strains as described previously [24]. In brief, each set of the 227 single-copy orthologous protein sequences obtained using OrthoFinder was first aligned with MAFFT [25] to the pre-computed, then a concatenated alignment of the proteins was generated with the command “genome2cpAAI.py”; finally, the cpAAI values were calculated by a custom R script from the ‘ape’ package [26]. The numerical distribution of AAI, ANI and cpAAI were visualized by using R package ggplot2 (Version: 3.5.0).

### 2.5. Chemotaxonomy and Physiology

The four novel strains were characterized through a series of chemotaxonomic and physiological analyses to determine its biochemical profile and assess its growth conditions. Strains were cultured in tryptic soy broth (Difco) for 24 h at 30 °C. Harvested cells were analyzed for polar lipids and respiratory quinones following established protocols [27,28,29,30]. The extraction of cellular fatty acids followed the method by Kuykendall [31] and then analyzed with the Sherlock Microbial Identification System (MIDI) [32].

Growth conditions, including pH, temperature and salinity, were optimized following methods by Li et al. [33]. Growth temperatures ranged from 4 °C to 50 °C (intervals: 4, 10, 15, 20, 25, 28, 30, 37, 41, 45 and 50 °C). pH was adjusted between 4 and 11 using specific buffers [34,35]: Na_2_HPO4/NaOH (pH 10.0–11.0), tris (pH 8.0–9.0), phosphate (pH 6.0–7.0) and citrate/Na_2_HPO4 (pH 4.0–5.0). Salinity effects were tested at 1–9% (*w*/*v*, 1% intervals). Gram staining followed Jenkins’ protocol [36]. An anaerobic jar was used to assess the anaerobic growth of the four novel strains over one week [33]. Catalase and oxidase activities were evaluated via methods from Smibert and Krieg [37]. The assessment of carbon source utilization, enzyme activity and acid production was conducted using API-ZYM, API 20NE and API 50 CH kits (bioMérieux, Craponne, France).

## 3. Results

**Figure 1:**
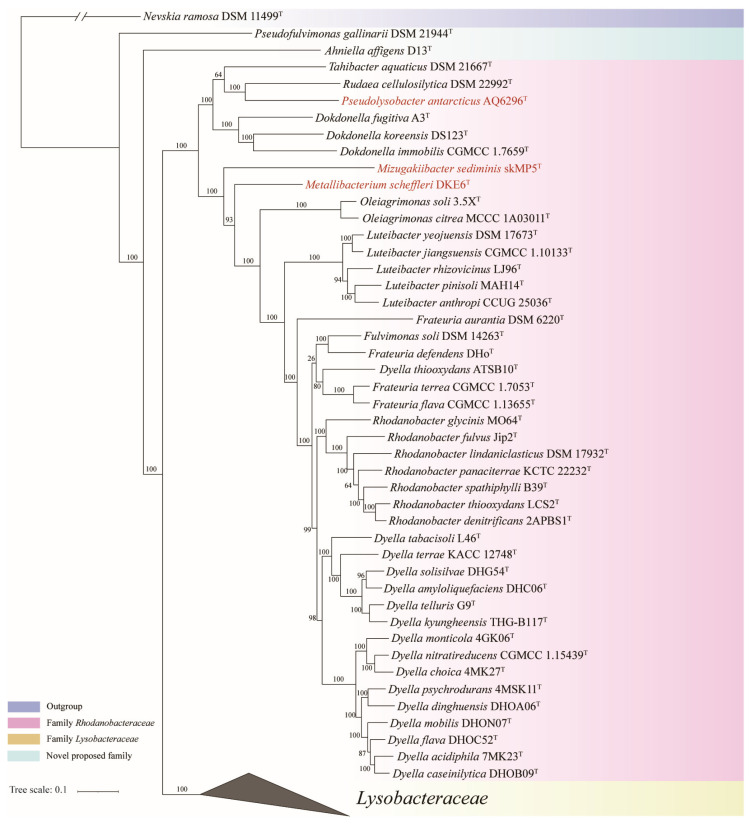
The first part of the concatenated protein phylogenetic tree among strains in order *Lysobacterales* based on a concatenated alignment of 227 ubiquitous single-copy proteins, focusing on family *Rhodanobacteraceae*. For details and abbreviations see Figure S1. The scale bar corresponds to 0.1 substitutions per amino acid position.

**Figure 2:**
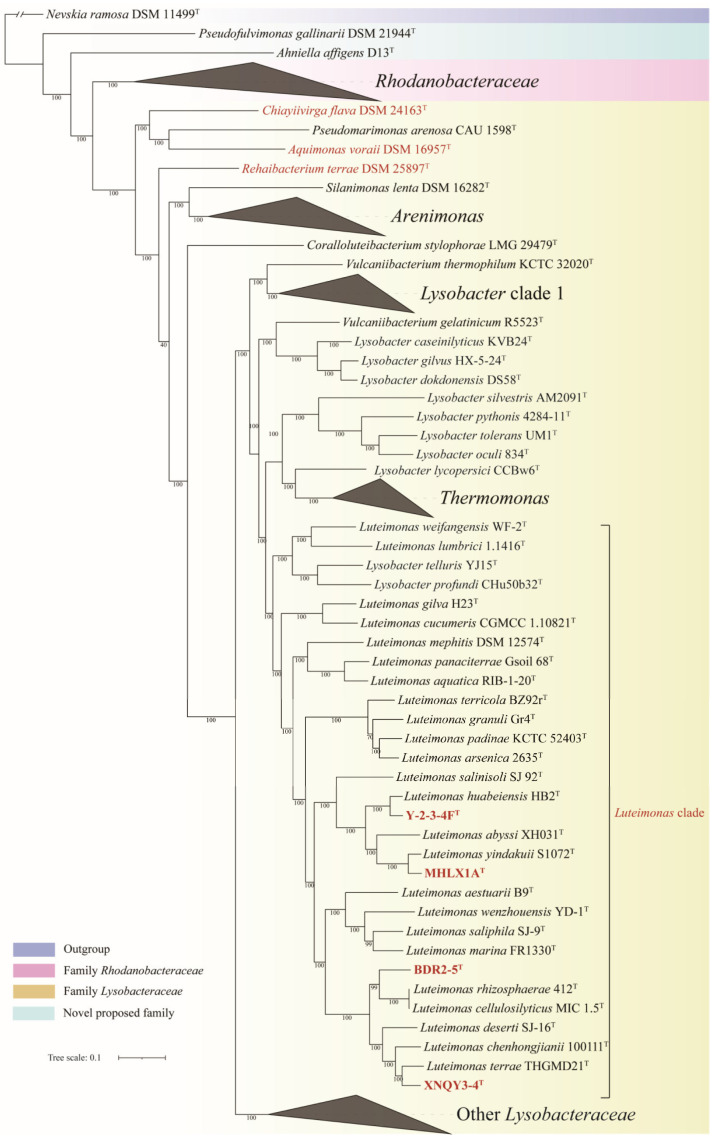
The second part of the concatenated protein phylogenetic tree among strains in order *Lysobacterales* based on a concatenated alignment of 227 ubiquitous single-copy proteins, focusing on part of family *Lysobacteraceae*. For details and abbreviations see Figure S1. The scale bar corresponds to 0.1 substitutions per amino acid position.

The concatenated proteins and UBCG phylogenetic trees exhibited a similar overall phylogenetic backbone, with most species in order *Lysobacterales* consistently clustering into similar monophyletic clades with high bootstrap support values. The phylogenetic trees based on concatenated proteins are shown in Figure 1 and Figure 2, while the full details of the concatenated proteins and UBCG phylogenetic trees were provided in Figures S1 and S2. The 16S rRNA gene phylogenetic tree is presented in Figure S3. However, it displayed low resolution, and the distribution of strains among closely related genera appeared amphibolous, consistent with previous findings [38]. Therefore, in the present study, genome-based phylogenetic trees were primarily used to infer the taxonomic relationships of species within the order *Lysobacterales*.

Until now, there has been no consensus on a standardized metric for family-level classification, but species within the same family are generally more similar to each other than to species from different families [39]. For example, genomic metrics such as AAI and ANI can serve as complementary tools for prokaryotic classification [40,41]. In addition to these metrics, cpAAI has recently been proposed as a reliable demarcation method among several prokaryotic families [42,43]. Therefore, in the present study, cpAAI values were also calculated as supplementary evidence for the reclassification of the order *Lysobacterales*.

### 3.1. Proposal for New Family

All phylogenetic trees (Figure 1 and Figure 2, Figures S1 and S2) consistently showed that the order *Lysobacterales* was divided into four monophyletic branches: two corresponding to the families *Rhodanobacteraceae* and *Lysobacteraceae* and two composed separately of *A. affigens* D13^T^ and *Pseudofulvimonas gallinarii* DSM 21944^T^. *A. affigens* D13^T^ and *P. gallinarii* DSM 21944^T^ formed two distinct, strongly supported branched outside of the clades of *Rhodanobacteraceae* and *Lysobacteraceae*, suggesting that they represent two novel families.

**Figure 3:**
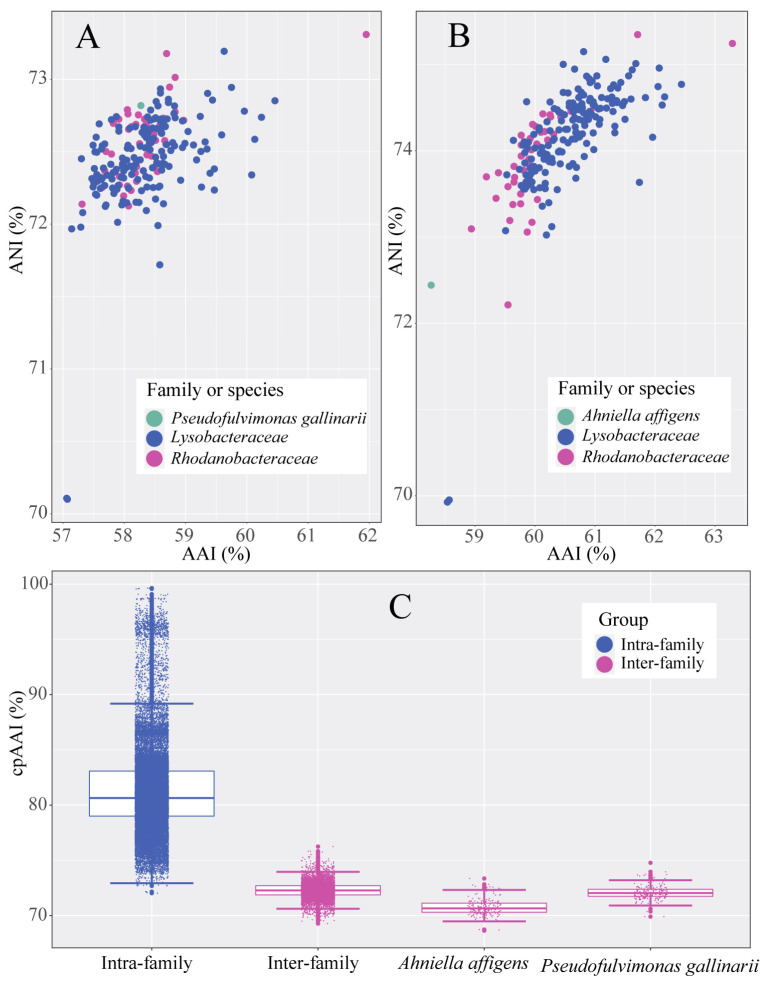
AAI and ANI distribution between *Ahniella affigens* D13^T^ and other members within order *Lysobacterales* are shown in plot (**A**); AAI and ANI distribution between *Pseudofulvimonas gallinarii* DSM 21944^T^ and other members within order *Lysobacterales* are shown in plot (**B**). Box plot indicates pairwise cpAAI values within and between families of order *Lysobacterales*; pairwise cpAAI values between *Ahniella affigens* D13^T^ and other members within order *Lysobacterales*; and pairwise cpAAI values between *Pseudofulvimonas gallinarii* DSM 21944^T^ and other members within order *Lysobacterales* (**C**).

**Figure 4:**
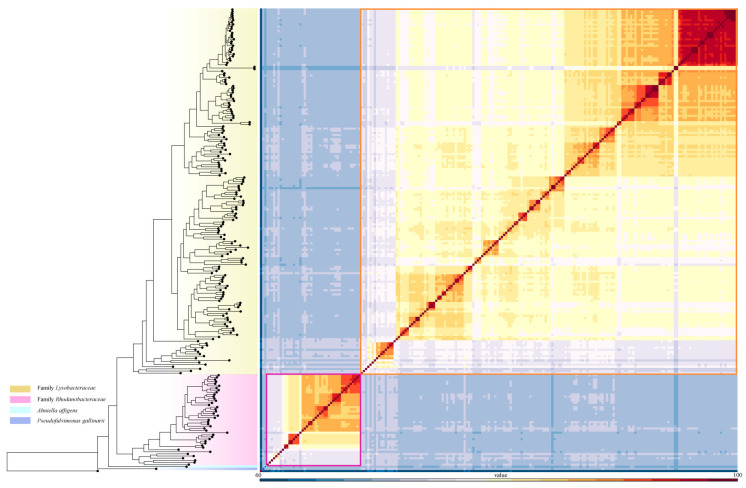
Heatmap of pairwise cpAAI within order *Lysobacterales*; the proposed genera are marked with quadrangles and the pairwise cpAAI values are provided in Table S1. The phylogenetic tree on the left of the plot is the concatenated protein tree, and different proposed families are shown in different colors.

AAI and ANI analyses (Figure 3A) also revealed that *P. gallinarii* DSM 21944^T^ did not cluster with any known families within *Lysobacterales*, supporting its placement into a novel family. The cpAAI results (Figure 3C) showed that intra-family and inter-family species could generally be clearly distinguished, although a few overlaps were observed, likely due to differences in evolutionary and ecological rates [39]. Specifically, *P. gallinarii* DSM 21944^T^ exhibited lower cpAAI values (69.09–74.78%) when compared with species from known families, even lower than the inter-family cpAAI values observed within *Lysobacterales* (72.03–99.65%) (Figure 3C and Figure 4).

Furthermore, according to the EzBioCloud database [44], *P. gallinarii* DSM 21944^T^ shared less than 93.4% 16S rRNA gene sequences similarity with all type strains in the order *Lysobacterales*, indicating that it cannot be assigned to any existing family within the order. According to the previous literature, the standardized 16S rRNA gene sequence threshold for defining novel families is typically 86.5% [45]. Although the similarity observed here is above this threshold, it still falls significantly below the common inter-family similarity range within *Lysobacterales*, suggesting substantial phylogenetic distinctiveness. Moreover, a recent study indicates that thresholds based on 16S rRNA gene sequences can overlap between taxa, and that higher-resolution genomic indices and comprehensive phylogenomic analysis are necessary for robust taxonomic delineation [46]. Therefore, based on all available data, we proposed that *P. gallinarii* DSM 21944^T^ should be classified into a novel family within the order *Lysobacterales*.

Similarly, AAI and ANI analyses (Figure 3B) showed that *A. affigens* D13^T^ did not belong to any known family within *Lysobacterales*, suggesting that it should also be placed into a novel family. The majority of cpAAI values between *A. affigens* D13^T^ and other members of *Lysobacterales* were also lower than the inter-family cpAAI values observed within the order (Figure 3C and Figure 4). These results corroborate recent findings that *A. affigens* D13^T^ belongs to a novel family [47].

Additionally, the polar lipids of *P. gallinarii* DSM 21944^T^ were diphosphatidylglycerol (DPG) and phosphatidylmethylethanolamine (PME), and the major fatty acids of *P. gallinarii* DSM 21944^T^ were iso-C_15:0_, iso-C_17:1_ *ω*9*c* and iso-C_17:0_. Significant differences in physicochemical properties between *P. gallinarii* DSM 21944^T^ and the type genera of existing families in *Lysobacterales* (Table S1) further support its classification into a separate family. In summary, we propose that *P. gallinarii* DSM 21944^T^ should represent a distinct novel family within the order *Lysobacterales*.

### 3.2. Proposal for the Transfer of Genera Between Families Lysobacteraceae and Rhodanobacteraceae

*Pseudolysobacter antarcticus* AQ6-296^T^, *Mizugakiibacter sediminis* skMP5^T^ and *Metallibacterium scheffleri* DKE6^T^, currently classified under *Lysobacteraceae*, were consistently clustered within the family *Rhodanobacteraceae* across all phylogenetic trees (Figure 1, Figures S1 and S2), suggesting that they should be reclassified into *Rhodanobacteraceae*. Similarly, *Chiayiivirga flava* DSM 24163^T^, *Aquimonas voraii* DSM 16957^T^ and *Rehaibacterium terrae* DSM 25897^T^, current placed under *Rhodanobacteraceae*, were clustered within *Lysobacteraceae* (Figure 2, Figures S1 and S2), indicating that they should be transferred to *Lysobacteraceae*. These reassignments are further supported by the heatmap of cpAAI values (Figure 4), which are consistent with the phylogenomic trees. Based on these findings, we propose the following reclassification that *P. antarcticus* AQ6-296^T^, *M. sediminis* skMP5^T^ *M. scheffleri* DKE6^T^ should be transferred to family *Rhodanobacteraceae* and *C. flava* DSM 24163^T^, *A. voraii* DSM 16957^T^ and *R. terrae* DSM 25897^T^ be transferred to family *Lysobacteraceae*.

### 3.3. Proposal for Four Novel Species

#### 3.3.1. Genome-Based Phylogenetic and Metrics Analyses

In the concatenated protein and UBCG phylogenetic trees (Figure 2, Figures S1 and S2), strains Y-2-3-4F^T^, MHLX1A^T^, BDR2-5^T^ and XNQY3-4^T^ formed distinct strongly branches within the genus *Luteimonas*. These trees suggested that the four strains represent four novel species within *Luteimonas*.

Based on 16S rRNA gene sequences similarity, strains Y-2-3-4F^T^ and MHLX1A^T^ were most closely related to *Luteimonas huabeiensis* HB2^T^ (99.0%) and *Luteimonas yindakui* S-1072^T^ (98.7%), respectively, consistent with the phylogenetic analyses. Strain BDR2-5^T^ was closely related to *Luteimonas chenhongjianii* 100111^T^ (98.3%) and *Luteimonas aestuarii* B9^T^ (98.2%), and strain XNQY3-4^T^ was closely related to *L. chenhongjianii* 100111^T^ (98.6%) and *Luteimonas terrae* HG-MD21^T^ (98.3%). ANI and dDDH values, golden standards for species delineation [6], were calculated between the four strains and their closest reference strains, with all results falling below the recommended species boundary thresholds (ANI, 95–96%, and dDDH, 70%) (Table 1). These results confirm that the four strains represent four novel species within the genus *Luteimonas*.

#### 3.3.2. Chemotaxonomic and Physiological Analysis

Although the polar lipids of the four strains shared several major components, including diphosphatidylglycerol (DPG), phosphatidyl ethanolamine (PE) and phosphatidylglycerol (PG), certain lipids (PL1, AL, APL1) were able to distinguish the strains from each other (Figure S4). Additionally, differences in the polar lipids profiles also separated these strains from related species within *Luteimonas*. The presence of four unidentified lipids (L) differentiated strain XNQY3-4^T^ from its relatives. Strain MHLX1A^T^ could be distinguished from *L. yindakuii* S-1072^T^ its higher PE content and absence of unidentified aminophospholipid (APL) and unidentified glycolipid (GL) could distinguish strain MHLX1A^T^ from *L. yindakuii* S-1072^T^. The sole respiratory quinone detected in all four strains was Q-8, which is consistent with their assignment to the genus *Luteimonas*.

The main fatty acids of strain Y-2-3-4F^T^ were similar to those of *L. huabeiensis* HB2^T^, but it had significantly higher proportions of iso-C_16:0_ and summed features 9. Strain MHLX1A^T^ exhibited notably greater levels of iso-C_15:0_ and iso-C_17:0_, and a lower amount of iso-C_16:0_ compared to its relatives. The absence of iso-C_10:0_ and higher levels of iso-C_15:0_ and iso-C_17:0_ distinguish strain BDR2-5^T^ from *L. chenhongjianii* 100111^T^ and *L. aestuarii* B9^T^. Likewise, strain XNQY3-4^T^ differed from *L. chenhongjianii* 100111^T^ and *L. terrae* THG-MD21^T^ by its larger amounts of summed features 9 and iso-C_17:0_ and lower levels of iso-C_10:0_ (Table S2).

On TSA medium, all four strains produced yellow, viscous colonies. Despite sharing some basic features, they differed in optimal growth pH, temperature and NaCl tolerance. They were also distinguishable from each other and from related species based on substrate utilization and enzyme activity profiles. The phenotypic characteristics are summarized in Table 2 and detailed in the strain descriptions. Altogether, these chemotaxonomic and physiological features confirmed that the four strains represent four novel species within the genus *Luteimonas*.

## 4. Discussion and Conclusions

The increasing availability of genomic data have provided a more comprehensive framework for understanding the taxonomy of families within the order *Lysobacterales*. Although genomic metrics such as AAI, ANI and POCP values [53,54] are not absolute standards for classifications above the genus level, higher whole-genome similarities are generally observed within genera or closely related groups [39,55]. In our analysis, the AAI, ANI and POCP values between the family *Marinicellaceae* and members of *Lysobacterales* were significantly lower than those between *Nevskiales* and *Lysobacterales*, suggesting that *Marinicellaceae* may belong to a different, albeit closely related, order of *Lysobacterales*. Moreover, the clustering of *Marinicellaceae* outside *Lysobacterales* in previous studies [3] supports its placement in a distinct order.

Because single-copy core genes can vary greatly among families or orders [24,43], and to ensure phylogenetic accuracy, *Marinicellaceae* was excluded from this study. We focused instead on two main families within *Lysobacterales*: *Lysobacteraceae* and *Rhodanobacteraceae*. Based on genome-based phylogenetic and phylogenomic analyses, our results support that the families within *Lysobacterales* are monophyletic. We propose that the order *Lysobacterales* should be divided into four families, *Ahniellaceae*, *Lysobacteraceae*, *Rhodanobacteraceae* and *Pseudofulvimonadaceae* (the latter proposed in this study).

The phylogenetic trees constructed using concatenated core proteins and UBCG markers also indicate that the taxonomy of several genera within *Lysobacterales* requires revision. The genus *Lysobacter* consistently separated into several well-supported clades, suggesting the need for recognition of new genera. A recent study proposed splitting *Lysobacter* into multiple genera based on AAI values and phylogenomic analyses, suggesting an AAI threshold of 69.5–76.0% for genus-level delimitation within the family *Lysobacteraceae*. However, previous research highlighted the limitations of AAI values in genus-level classification [24,52,53,54,55]. For example, AAI values within *Luteimonas* range from 69.0% to 90.1%, and the newly proposed genus *Cognatiluteimonas* shares AAI values of 69–73.9% with *Luteimonas* [3]. Therefore, additional evidence will be needed before formally reclassifying *Lysobacter*. The genera *Xanthomonas*, *Pseudoxanthomonas* and *Stenotrophomonas* may also require taxonomic revisions, and a recent study also confirmed that these three genera should merged into genus *Xanthomonas* [56]. Nevertheless, such comprehensive taxonomic revisions are beyond the scope of the current study.

A total of 218 genome sequences (217 *Lysobacterales* and 1 *Nevskiales*) were analyzed. Genome-based phylogenetic and phylogenomic metrics revealed the need to revise the family-level taxonomy within *Lysobacterales*. Phylogenetic analyses using UBCG and concatenated proteins, along with ANI, AAI and cpAAI values, demonstrated that *Pseudofulvimonas gallinarii* DSM 21944^T^ should be assigned to a distinct novel family, and that six genera should be reclassified across existing families. Furthermore, four novel species within the family *Lysobacteraceae* were identified based on phylogenetic analysis, physiological and biochemical characteristics and genomic data and were described in this study.

### 4.1. Taxonomic Levels: New Family

#### Description of *Pseudofulvimonadaceae* fam. nov.

*Pseu.do.ful.vi.mo.na.da’ce.ae* (N.L. fem. n. *Pseudofulvimonas*, type genus of the family; *-aceae*, ending to denote a family; N.L. fem. pl. n. *Pseudofulvimonadaceae*, the *Pseudofulvimonas* family).

Cells are Gram-strain-negative, aerobic, non-motile, rod-shaped. The predominant respiratory quinone is Q-8. The major cellular fatty acids are usually iso-C_15:0_, iso-C_17:1_ *ω*9*c* and iso-C_17:0_. The DNA G+C content is 67.6 mol %. The family is defined based on phylogenetic analyses of UBCG and concatenated protein phylogenetic trees, phylogenomic metric analyses of AAI, ANI and cpAAI. The description of the family is as given for *Pseudofulvimonas* [57], which is the type and currently the only genus of the family.

### 4.2. The Transfer for the Member of Family Rhodanobacteraceae and Lysobacteraceae

*Pseudolysobacter antarcticus*, *Mizugakiibacter sediminis* and *Metallibacterium scheffleri* were transferred to family *Rhodanobacteraceae*, and *Chiayiivirga flava*, *Aquimonas voraii*, *Rehaibacterium terrae* were transferred to family *Lysobacteraceae*.

### 4.3. Taxonomic Levels: New Species

#### 4.3.1. Description of *Alterluteimonas quercicellularis* sp. nov.

*Alterluteimonas quercicellularis* (quer.ci.cel.lu.la’ris. N.L. gen. fem. n. *quercus*, of quercus tree; L. fem. n. *cellula*, cell; N.L. fem. n. *quercicellularis*, a bacterium isolated from quercus tree).

Cells are Gram-stain-negative, aerobic, motile with a single polar flagellum, catalase- and oxidase-positive, rod-shaped, 1.2–1.8 mm in length and 0.6–0.8 mm in width. Colonies are yellow, circular, viscous, smooth, with entire margins after incubation for 2 days at 28 °C on TSA. The strain can grow at 10–41 °C (optimum, 28–30 °C) at pH 6–9 (optimum, pH 7–8). Growth occurs at a concentration of 0–5% (*w*/*v*) NaCl. Positive for alkaline phosphatase, leucine arylamidase, trypsin, *α*-chymotrypsin, acid phosphatase, naphthol-AS-BI-phosphohydrolase, *α*-glucosidase; weakly positive for esterase lipase (C8), esterase (C4), lipase (C14), valine arylamidase, *β*-galactosidase, *β*-glucosidase; negative for N-acetyl-*β*-glucosaminidase, cystine arylamidase, *α*-galactosidase, *β*-glucuronidase, *α*-mannosidase, *α*-fucosidase (API ZYM). Positive for reduction of nitrates to nitrites, esculin, gelatin hydrolysis, N-acetyl-glucosamine, D-maltose, malic acid; weakly positive for D-glucose, D-mannose; and negative for the rest tests in API 20NE.

Positive results in tests of using the following carbon sources: D-glucose, amygdalin, esculin ferric citrate, D-cellobiose, D-maltose, D-trehalose, D-melezitose, glycogen; weakly positive for D-xylose, D-lactose, starch, gentiobiose and D-lyxose, and negative for the rest tests in API 50 CH. The polar lipids were DPG, PE, PG and four unidentified lipids (L). The respiratory quinones were Q-8. The predominant fatty acids were iso-C_15:0_, iso-C_16:0_ and summed features 3 (C_16:1_ *ω*7*c* and/or C_16:1_ *ω*6*c*). The type strain is Y-2-3-4F^T^ (= CFCC 15605^T^ = LMG 32557^T^), isolated from Ningxia, China. The strain Y-2-3-4F^T^ was predicted to have 3911 coding genes, 52 tRNA genes, 3 rRNA genes and 7 other RNA genes, and the DNA G+C content was 73.0 mol %.

#### 4.3.2. Description of *Alterluteimonas muca* sp. nov.

*Alterluteimonas muca* (mu’ca. L. fem. adj. *muca*, mucus, referring to the characteristics of the colonies).

Cells are Gram-stain-negative, aerobic, motile with a single polar flagellum, catalase- and oxidase-positive, rod-shaped, 1.5–2.0 mm in length and 0.5–0.8 mm in width. Colonies are pale yellow, circular, viscous, smooth, with entire margins after incubation for 2 days at 28 °C on TSA. The strain can grow at 10–37 °C (optimum, 28 °C) at pH 6–8.5 (optimum, pH 7–7.5). Growth occurs at a concentration of 0–4% (*w*/*v*) NaCl. Positive for alkaline phosphatase, esterase lipase (C8), esterase (C4), leucine arylamidase, valine arylamidase, trypsin, *α*-chymotrypsin, acid phosphatase, naphthol-AS-BI-phosphohydrolase; weakly positive for lipase (C14) and cystine arylamidase; negative for the rest tests in API ZYM. Positive for esculin, gelatin hydrolysis, and negative for the rest tests in API 20NE. Positive results in tests of using the following carbon sources: D-glucose, D-fructose, D-mannose, salicin, D-lactose, D-melibiose and D-trehalose, and negative for the rest tests in API 50 CH. The polar lipids were DPG, PE, PG, PL1, AL and four unidentified lipids (L). The respiratory quinones were Q-8. The predominant fatty acids were iso-C_15:0_, iso-C_17:0_ and summed features 3 (C_16:1_ *ω*7*c* and/or C_16:1_ *ω*6*c*). The type strain is MHLX1A^T^ (= CFCC 16400^T^ = LMG 32554^T^), isolated from Beijing, China. The strain MHLX1A^T^ was predicted to have 3009 coding genes, 45 tRNA genes, 3 rRNA genes and 4 other RNA genes, and the DNA G+C content was 69.1 mol %.

#### 4.3.3. Description of *Proluteimonas luteida* sp. nov.

*Proluteimonas luteida* (Lu’tei.da. L. fem. adj. *luteida*, yellow, referring to the color of the colonies).

Cells are Gram-stain-negative, aerobic, motile with a single polar flagellum, catalase- and oxidase-positive, rod-shaped, 1.5–1.8 mm in length and 0.5–0.7 mm in width. Colonies are yellow, moist, viscid with irregular margins after incubation for 2 days at 28 °C on TSA. The strain can grow at 10–37 °C (optimum, 28 °C) at pH 6–8.5 (optimum, pH 7–7.5). Growth occurs at a concentration of 0–4% (*w*/*v*) NaCl. Positive for alkaline phosphatase, esterase lipase (C8), esterase (C4), leucine arylamidase, trypsin, *α*-chymotrypsin, acid phosphatase and naphthol-AS-BI-phosphohydrolase; weakly positive for valine arylamidase; and negative for the rest tests in API ZYM. Positive for esculin, gelatin hydrolysis and malic acid; weakly positive for D-glucose assimilation and D-mannose; and negative for the rest tests in API 20NE. Positive results in tests of using the following carbon sources: salicin and negative for the rest tests in API 50 CH. The polar lipids were DPG, PE, PG, PL1, APL1 and two unidentified lipids (L). The respiratory quinones were Q-8. The predominant fatty acids were iso-C_15:0_, iso-C_17:0_ and summed features 3 (C_16:1_ *ω*7*c* and/or C_16:1_ *ω*6*c*). The type strain is BDR2-5^T^ (= CFCC 16401^T^ = LMG 32545^T^), isolated from Anhui, China. The strain BDR2-5^T^ was predicted to have 3520 coding genes, 20 tRNA genes, 7 rRNA genes and 5 other RNA genes, and the DNA G+C content was 69.7 mol %.

#### 4.3.4. Description of *Proluteimonas flavola* sp. nov.

*Proluteimonas flavola* (fla.vo.la. L. masc. adj. *flavus*, yellow; L. fem. adj. *flavola*, referring to the color of the colonies).

Cells are Gram-stain-negative, aerobic, motile with a single polar flagellum, catalase- and oxidase-positive, rod-shaped, 1.6–2.0 mm in length and 0.6–0.8 mm in width. Colonies are yellow, circular, viscous, smooth, with entire margins after incubation for 2 days at 28 °C on TSA. The strain can grow at 10–37 °C (optimum, 25–28 °C) at pH 6–9 (optimum, pH 7–8). Growth occurs at a concentration of 0–3% (*w*/*v*) NaCl. Positive for alkaline phosphatase, esterase lipase (C8), esterase (C4), leucine arylamidase, valine arylamidase, trypsin, α-chymotrypsin, acid phosphatase and naphthol-AS-BI-phosphohydrolase; negative for the rest tests in API ZYM. Positive for esculin, gelatin hydrolysis, D-maltose and malic acid; weakly positive for D-glucose assimilation; and negative for the rest tests in API 20NE. Positive results in tests of using the following carbon sources: D-fructose, salicin, D-maltose, D-lactose, D-melibiose and D-turanose, and negative for the rest tests in API 50 CH. The polar lipids were DPG, PE, PG, PL and four unidentified lipids (L). The respiratory quinones were Q-8. The predominant fatty acids were iso-C_15:0_, iso-C_16:0_, iso-C_17:0_ and summed features 3 (C_16:1_ *ω*7*c* and/or C_16:1_ *ω*6*c*). The type strain is XNQY3-4^T^ (= CFCC 16543^T^ = LMG 32556^T^), isolated from Qinghai, China. The strain XNQY3-4^T^ was predicted to have 3648 coding genes, 48 tRNA genes, 5 rRNA genes and 4 other RNA genes, and the DNA G+C content was 68.3 mol %.

## Figures and Tables

**Table 1:** Digital DNA–DNA hybridization (dDDH) values, average nucleotide identity (ANI) among the four novel strains and closely related type strains.

Strain	Y-2-3-4F^T^		MHLX1A^T^		BDR2-5^T^		XNQY3-4^T^	
	ANI	dDDH	ANI	dDDH	ANI	dDDH	ANI	dDDH
MHLX1A^T^	81.52	24.5						
BDR2-5^T^	79.43	22.9	77.84	21.6				
XNQY3-4^T^	78.07	21.6	77.92	21.3	82.20	25.7		
*Luteimonas huabeiensis* HB2^T^	90.78	41.9	87.27	24.2	79.50	22.7	77.85	21.6
*Luteimonas yindakuii* S-1072^T^	81.74	24.5	87.73	34.1	77.94	21.8	77.24	21.2
*Luteimonas chenhongjianii* 100111^T^	77.42	21.2	76.97	20.8	81.17	24.2	84.25	28.0
*Luteimonas terrae* THG-MD21^T^	77.80	21.5	76.61	20.8	81.55	24.6	88.53	30.0
*Luteimonas aestuarii* B9^T^	77.92	21.8	77.32	21.4	78.20	22.0	77.30	21.2

**Table 2:** Differential characteristics of the four novel strains and closely related type strains. Strains: 1, Y-2-3-4F^T^; 2, MHLX1A^T^; 3, BDR2-5^T^; 4, XNQY3-4^T^; 5, *Luteimonas huabeiensis* HB2^T^; 6, *Luteimonas yindakuii* S-1072^T^; 7, *Luteimonas chenhongjianii* 100111^T^; 8, *Luteimonas terrae* THG-MD21^T^; 9, *Luteimonas aestuarii* B9^T^. Data in column 5 are from [48], data in column 6 are from [49], data in column 7 are from [50], data in column 8 are from [51] and data in column 9 are from [52]. +, positive; −, negative; W, weakly positive.

Characteristic	1	2	3	4	5	6	7	8	9
Cell size (µm)	0.6–0.8 × 1.2–1.8	0.5–0.8 × 1.5–2	0.5–0.7 × 1.5–1.8	0.6–0.8 × 1.6–2.0	0.4–0.5 × 0.9–1.6	0.7–1 × 1.7–2.8	1.1–1.4 × 1.5–1.8	0.4–0.5 × 1.1–1.7	0.5 × 1.5–2.0
pH (optimum pH)	6–9 (7–8)	6–8.5 (7–7.5)	6–8.5 (7–7.5)	6–9 (7–8)	6–11 (7)	6.5–9.5 (7)	6–10 (7–8)	6.5–8 (7–7.5)	6.5–11 (8)
Temperature (Optimum temperature °C)	10–41 (28–30)	10–37 (28)	10–37 (28)	10–37 (25–28)	20–45 (30)	4–40 (28)	22–40 (35–37)	4–45 (25–30)	15–40 (34–37)
NaCl range (%, *w*/*v*)	0–5	0–4	0–4	0–3	0–5	0–2.5	0–3.5	0–5.5	0–4
Reduction of nitrate	+	−	−	−	−	−	−	+	−
Utilization of:									
D-mannose	−	+	−	−	+	−	−	−	+
D-mannitol	−	−	−	−	+	−	−	−	+
Methyl *α*-D-mannopyranoside	−	−	−	−	−	−	−	+	−
D-maltose	+	−	−	+	+	−	+	+	+
Glycogen	+	−	−	−	−	−	−	+	W
Xylitol	−	−	−	−	−	−	−	+	−
Enzyme activities:									
Esterase (C4)	W	+	+	+	+	+	−	+	W
Lipase (C14)	W	W	−	−	+	−	−	−	−
Valine arylamidase	W	+	W	+	+	−	−	+	+
Cystine arylamidase	−	W	−	−	+	−	−	W	W
Trypsin	+	+	+	+	+	−	+	−	+
*α*-chymotrypsin	+	+	+	+	+	+	−	+	+
*α*-glucosidase	+	−	−	−	−	−	−	+	W
N-acetyl-*β*-glucosaminidase	−	−	−	−	+	−	−	+	+
Hydrolysis from:									
Urease	−	−	−	−	+	−	−	+	+
Aesculin	+	+	+	+	+	+	−	+	+
Gelatin	+	+	+	+	+	−	−	+	+
G+C content (mol %)	73.0	69.1	69.7	68.3	67.0	69.2	68.3	64.4	64.7

## Data Availability

The GenBank/EMBL/DDBJ accession numbers for the 16S rRNA gene are OK287346–OK287349, and for the genome sequences of strains BDR2-5^T^, XNQY3-4^T^, Y-2-3-4F^T^ and MHLX1A^T^ are JAIWPT000000000, JAIWPU000000000, JAIWPR000000000 and JAIWPS000000000, respectively.

## Supplementary Materials

The following supporting information can be downloaded at: https://www.mdpi.com/article/10.3390/microorganisms13061212/s1. Table S1: the pairwise values of AAI, ANI and cpAAI and phenotypic comparisons within the species. Supplementary File S1: Supplementary Figures S1–S5 and Table S2.

## Abbreviations

The following abbreviations are used in this manuscript:

AAI	average amino acid identity
ANI	average nucleotide identity
cpAAI	core-proteome average amino acid identity
dDDH	digital DNA–DNA hybridization
