# Supplementary material for: Familial Reclassification Within Order Lysobacterales and Proposal of Four Novel Species
Source: Microorganisms. 2025 May 26;13(6):1212. doi: 10.3390/microorganisms13061212 (PMC12195097; doi:10.3390/microorganisms13061212)
Supplement: Supplementary file 1 [file microorganisms-13-01212-s001.zip › Supplementary file S1.pdf]

Familial reclassification within order *Lysobacterales* and proposal of four novel species

Supplementary Figures and Table S2

1. Concatenated proteins phylogenetic tree (Figure S1) 5
2. UBCG phylogenetic tree (Figure S2).. 10
3. 16S rRNA gene phylogenetic tree (Figure S3).. 16
4. Polar lipid profiles of the four novel strains (Figure S4) 17
5. Electron microscopy images of the four novel strains (Figure S5) 17
6. Cellular fatty acid profiles of the four strains and closely related type strains(Table S2).... 18

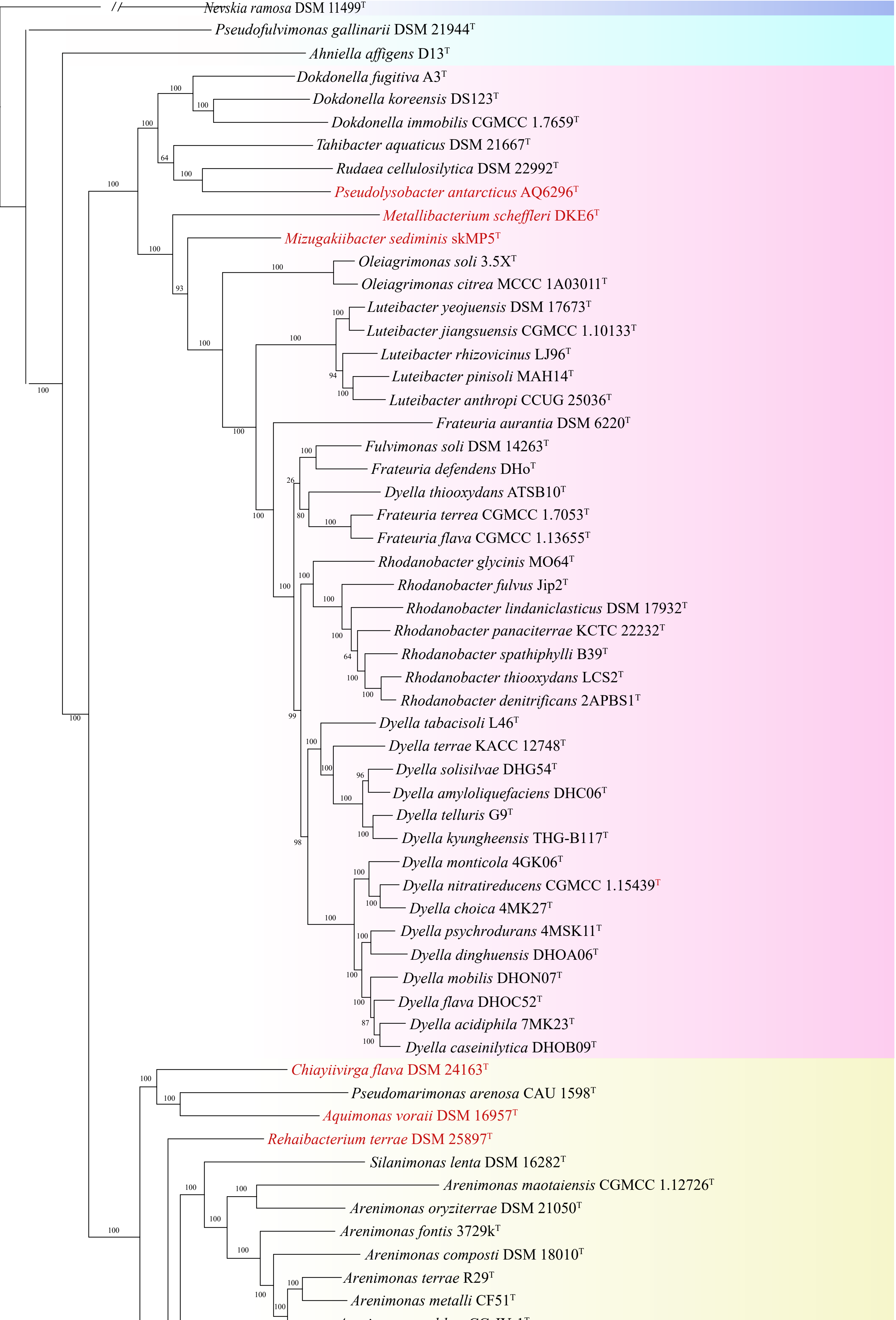
Nevskia ramosa DSM 11499T
Pseudofulvimonas gallinarii DSM 21944T
Ahniella affigens D13T
Dokdonella fugitiva A3T
Dokdonella koreensis DS123T
Dokdonella immobilis CGMCC 1.7659T
Tahibacter aquaticus DSM 21667T
Rudaea cellulosilytica DSM 22992T
Pseudolysobacter antarcticus AQ6296T
Metallibacterium scheffleri DKE6T
Mizugakiibacter sediminis skMP5T
Oleiagrimonas soli 3.5XT
Oleiagrimonas citrea MCCC 1A03011T
Luteibacter yeojuensis DSM 17673T
Luteibacter jiangsuensis CGMCC 1.10133T
Luteibacter rhizovicinus LJ96T
Luteibacter pinisoli MAH14T
Luteibacter anthropi CCUG 25036T
Frateuria aurantia DSM 6220T
Fulvimonas soli DSM 14263T
Frateuria defendens DHoT
Dyella thiooxydans ATSB10T
Frateuria terrea CGMCC 1.7053T
Frateuria flava CGMCC 1.13655T
Rhodanobacter glycinis MO64T
Rhodanobacter fulvus Jip2T
Rhodanobacter lindaniclasticus DSM 17932T
Rhodanobacter panaciterrae KCTC 22232T
Rhodanobacter spathiphylli B39T
Rhodanobacter thiooxydans LCS2T
Rhodanobacter denitrificans 2APBS1T
Dyella tabacisoli L46T
Dyella terrae KACC 12748T
Dyella solisilvae DHG54T
Dyella amyloliquefaciens DHC06T
Dyella telluris G9T
Dyella kyungheensis THG-B117T
Dyella monticola 4GK06T
Dyella nitratireducens CGMCC 1.15439T
Dyella choica 4MK27T
Dyella psychrodurans 4MSK11T
Dyella dinghuensis DHOA06T
Dyella mobilis DHON07T
Dyella flava DHOC52T
Dyella acidiphila 7MK23T
Dyella caseinilytica DHOB09T
Chiayiivirga flava DSM 24163T
Pseudomarimonas arenosa CAU 1598T
Aquimonas voraii DSM 16957T
Rehaibacterium terrae DSM 25897T
Silanimonas lenta DSM 16282T
Arenimonas maotaiensis CGMCC 1.12726T
Arenimonas oryziterrae DSM 21050T
Arenimonas fontis 3729kT
Arenimonas composti DSM 18010T
Arenimonas terrae R29T
Arenimonas metalli CF51T

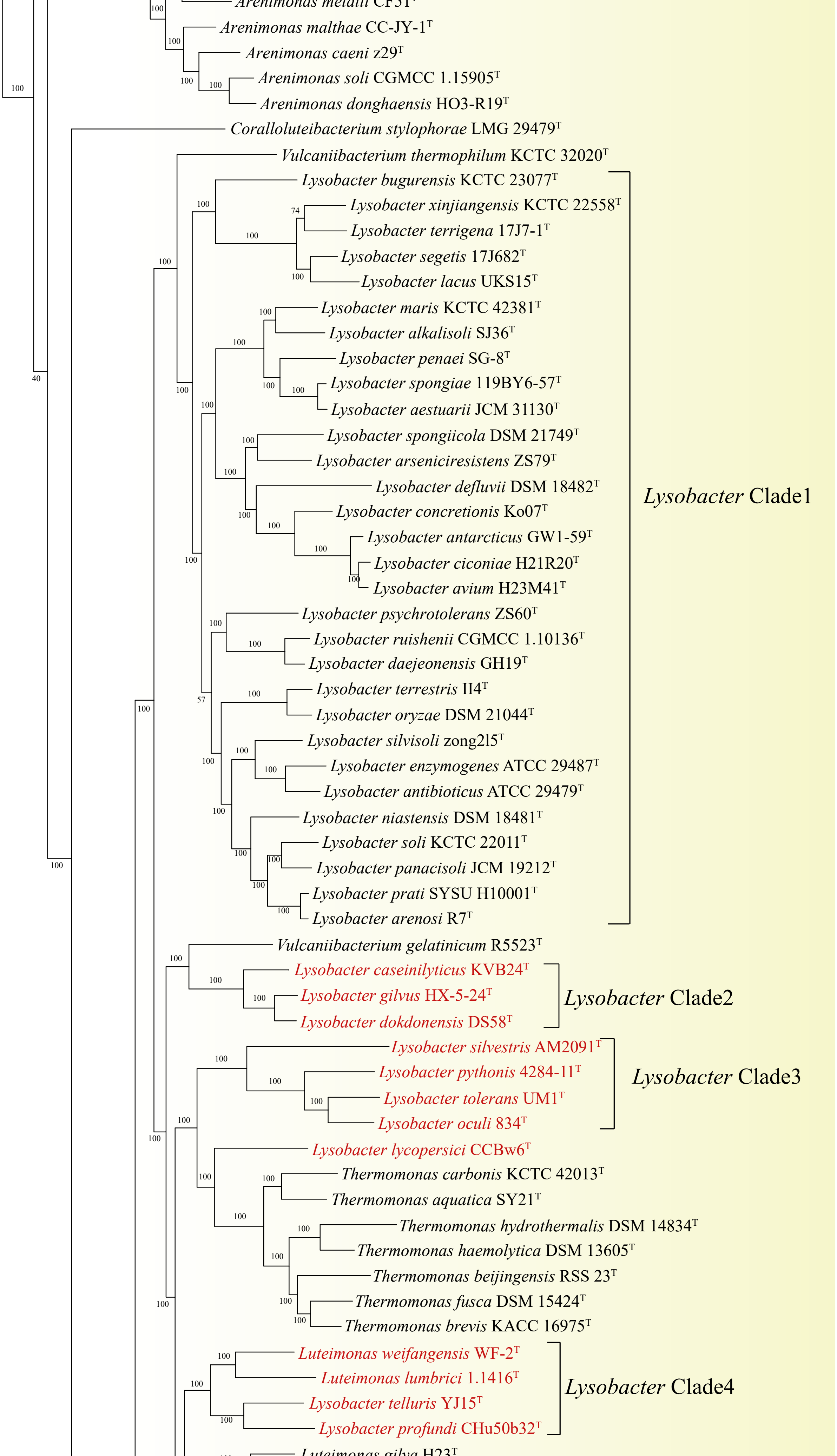
Arenimonas metalli CF5-1T
Arenimonas malthae CC-JY-1T
Arenimonas caeni z29T
Arenimonas soli CGMCC 1.15905T
Arenimonas donghaensis HO3-R19T
Coralloluteibacterium stylophorae LMG 29479T
Vulcaniibacterium thermophilum KCTC 32020T
Lysobacter bugurensis KCTC 23077T
Lysobacter xinjiangensis KCTC 22558T
Lysobacter terrigena 17J7-1T
Lysobacter segetis 17J682T
Lysobacter lacus UKS15T
Lysobacter maris KCTC 42381T
Lysobacter alkalisoli SJ36T
Lysobacter penaei SG-8T
Lysobacter spongiae 119BY6-57T
Lysobacter aestuarii JCM 31130T
Lysobacter spongiicola DSM 21749T
Lysobacter arseniciresistens ZS79T
Lysobacter defluvii DSM 18482T
Lysobacter concretionis Ko07T
Lysobacter antarcticus GW1-59T
Lysobacter ciconiae H21R20T
Lysobacter avium H23M41T
Lysobacter psychrotolerans ZS60T
Lysobacter ruishenii CGMCC 1.10136T
Lysobacter daejeonensis GH19T
Lysobacter terrestris II4T
Lysobacter oryzae DSM 21044T
Lysobacter silvisoli zong2l5T
Lysobacter enzymogenes ATCC 29487T
Lysobacter antibioticus ATCC 29479T
Lysobacter niastensis DSM 18481T
Lysobacter soli KCTC 22011T
Lysobacter panacisoli JCM 19212T
Lysobacter prati SYSU H10001T
Lysobacter arenosi R7T
Lysobacter Clade1
Vulcaniibacterium gelatinicum R5523T
Lysobacter caseinilyticus KVB24T
Lysobacter gilvus HX-5-24T
Lysobacter dokdonensis DS58T
Lysobacter Clade2
Lysobacter silvestris AM2091T
Lysobacter pythonis 4284-11T
Lysobacter tolerans UM1T
Lysobacter oculi 834T
Lysobacter Clade3
Lysobacter lycopersici CCBw6T
Thermomonas carbonis KCTC 42013T
Thermomonas aquatica SY21T
Thermomonas hydrothermalis DSM 14834T
Thermomonas haemolytica DSM 13605T
Thermomonas beijingensis RSS 23T
Thermomonas fusca DSM 15424T
Thermomonas brevis KACC 16975T
Luteimonas weifangensis WF-2T
Luteimonas lumbrici 1.1416T
Lysobacter telluris YJ15T
Lysobacter profundi CHu50b32T
Lysobacter Clade4
Luteimonas gilva H23T

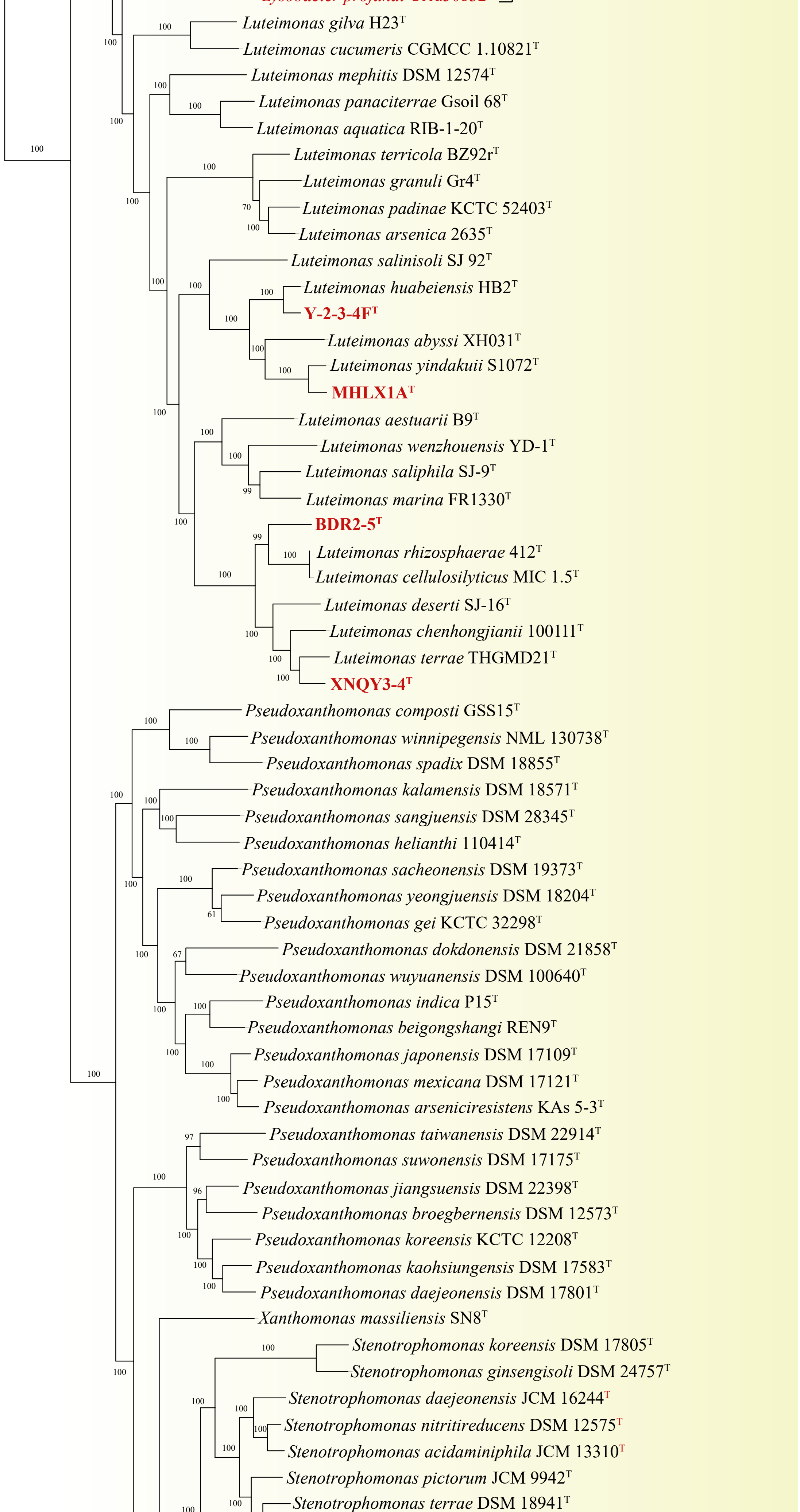
Luteimonas gilva H23T
Luteimonas cucumeris CGMCC 1.10821T
Luteimonas mephitis DSM 12574T
Luteimonas panaciterrae Gsoil 68T
Luteimonas aquatica RIB-1-20T
Luteimonas terricola BZ92rT
Luteimonas granuli Gr4T
Luteimonas padinae KCTC 52403T
Luteimonas arsenica 2635T
Luteimonas salinisoli SJ 92T
Luteimonas huabeiensis HB2T
Y-2-3-4FT
Luteimonas abyssi XH031T
Luteimonas yindakuii S1072T
MHLX1AT
Luteimonas aestuarii B9T
Luteimonas wenzhouensis YD-1T
Luteimonas saliphila SJ-9T
Luteimonas marina FR1330T
BDR2-5T
Luteimonas rhizosphaerae 412T
Luteimonas cellulosilyticus MIC 1.5T
Luteimonas deserti SJ-16T
Luteimonas chenhongjianii 100111T
Luteimonas terrae THGMD21T
XNQY3-4T
Pseudoxanthomonas composti GSS15T
Pseudoxanthomonas winnipegensis NML 130738T
Pseudoxanthomonas spadix DSM 18855T
Pseudoxanthomonas kalamensis DSM 18571T
Pseudoxanthomonas sangjuensis DSM 28345T
Pseudoxanthomonas helianthi 110414T
Pseudoxanthomonas sacheonensis DSM 19373T
Pseudoxanthomonas yeongjuensis DSM 18204T
Pseudoxanthomonas gei KCTC 32298T
Pseudoxanthomonas dokdonensis DSM 21858T
Pseudoxanthomonas wuyuanensis DSM 100640T
Pseudoxanthomonas indica P15T
Pseudoxanthomonas beigongshangi REN9T
Pseudoxanthomonas japonensis DSM 17109T
Pseudoxanthomonas mexicana DSM 17121T
Pseudoxanthomonas arseniciresistens KAs 5-3T
Pseudoxanthomonas taiwanensis DSM 22914T
Pseudoxanthomonas suwonensis DSM 17175T
Pseudoxanthomonas jiangsuensis DSM 22398T
Pseudoxanthomonas broegbernensis DSM 12573T
Pseudoxanthomonas koreensis KCTC 12208T
Pseudoxanthomonas kaohsiungensis DSM 17583T
Pseudoxanthomonas daejeonensis DSM 17801T
Xanthomonas massiliensis SN8T
Stenotrophomonas koreensis DSM 17805T
Stenotrophomonas ginsengisoli DSM 24757T
Stenotrophomonas daejeonensis JCM 16244T
Stenotrophomonas nitritireducens DSM 12575T
Stenotrophomonas acidaminiphila JCM 13310T
Stenotrophomonas pictorum JCM 9942T
Stenotrophomonas terrae DSM 18941T

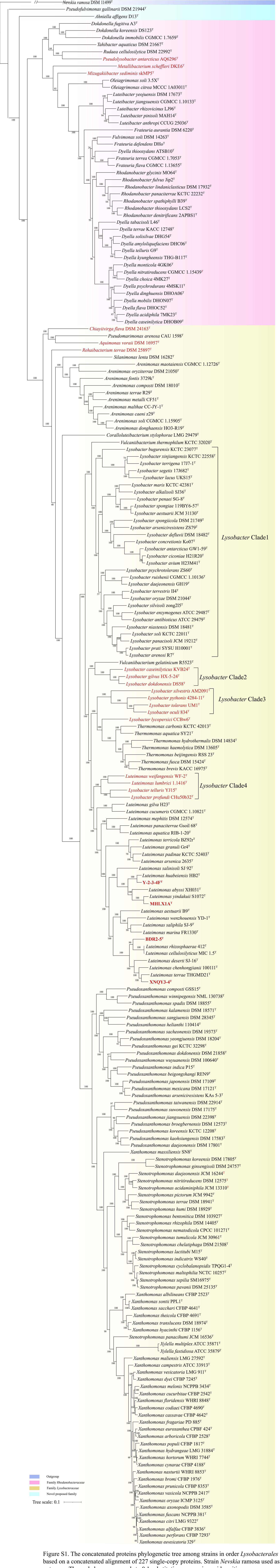

Figure S1. The concatenated proteins phylogenetic tree among strains in order *Lysobacterales* based on a concatenated alignment of 227 single-copy proteins. Strain *Nevskia* ramosa used as outgroup. The scale bar corresponds to 0.1 substitutions per amino acid position.

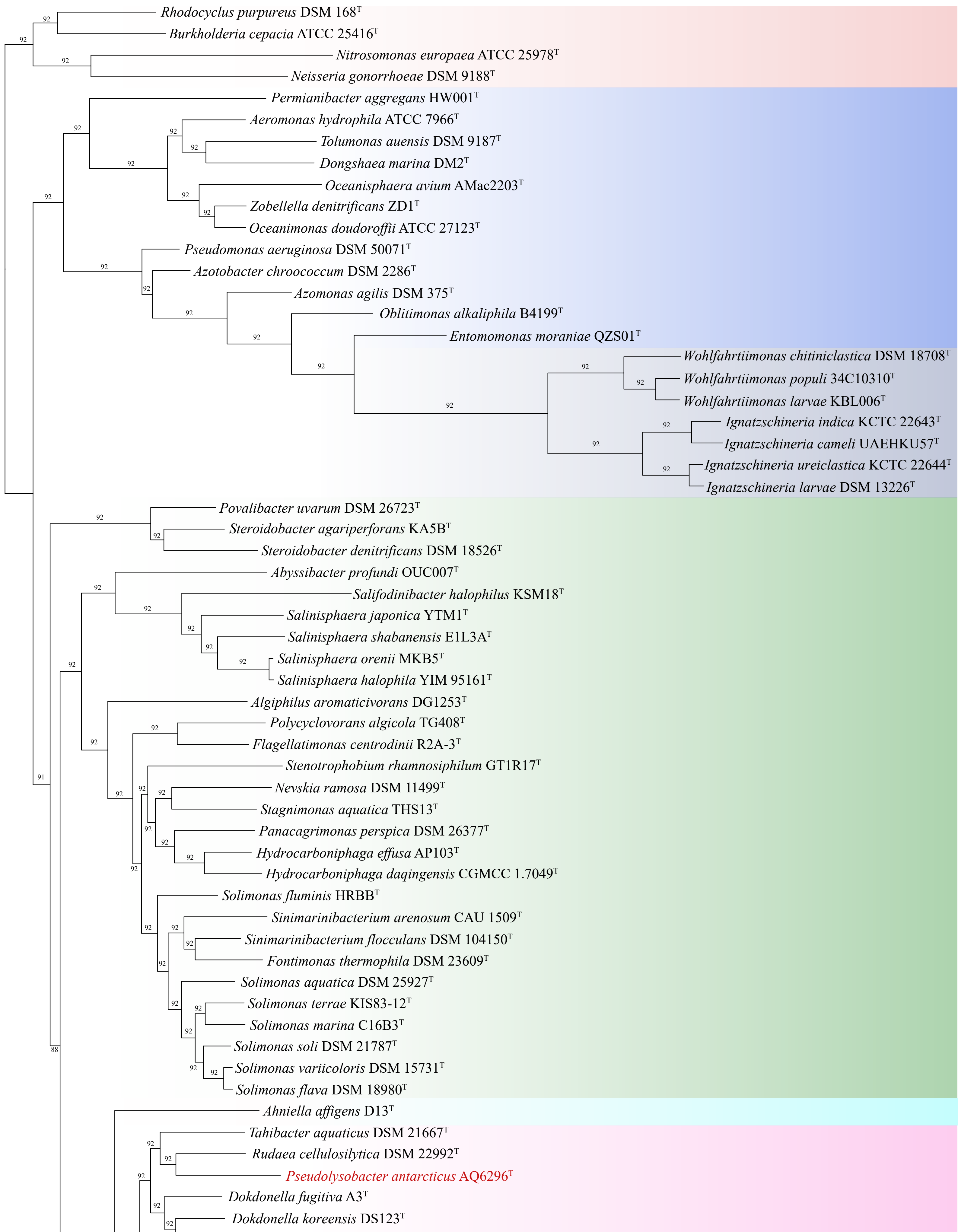
Rhodocyclus purpureus DSM 168T
Burkholderia cepacia ATCC 25416T
Nitrosomonas europaea ATCC 25978T
Neisseria gonorrhoeae DSM 9188T
Permianibacter aggregans HW001T
Aeromonas hydrophila ATCC 7966T
Tolumonas auensis DSM 9187T
Dongshaea marina DM2T
Oceanisphaera avium AMac2203T
Zobellella denitrificans ZD1T
Oceanimonas doudoroffii ATCC 27123T
Pseudomonas aeruginosa DSM 50071T
Azotobacter chroococcum DSM 2286T
Azomonas agilis DSM 375T
Oblitimonas alkaliphila B4199T
Entomomonas moraniae QZS01T
Wohlfahrtiimonas chitiniclastica DSM 18708T
Wohlfahrtiimonas populi 34C10310T
Wohlfahrtiimonas larvae KBL006T
Ignatzschineria indica KCTC 22643T
Ignatzschineria cameli UAEHKU57T
Ignatzschineria ureiclastica KCTC 22644T
Ignatzschineria larvae DSM 13226T
Povalibacter uvarum DSM 26723T
Steroidobacter agariperforans KA5BT
Steroidobacter denitrificans DSM 18526T
Abyssibacter profundi OUC007T
Salifodinibacter halophilus KSM18T
Salinisphaera japonica YTM1T
Salinisphaera shabanensis E1L3AT
Salinisphaera orenii MKB5T
Salinisphaera halophila YIM 95161T
Algiphilus aromaticivorans DG1253T
Polycyclovorans algicola TG408T
Flagellatimonas centrodinii R2A-3T
Stenotrophobium rhamnosiphilum GT1R17T
Nevskia ramosa DSM 11499T
Stagnimonas aquatica THS13T
Panacagrimonas perspica DSM 26377T
Hydrocarboniphaga effusa AP103T
Hydrocarboniphaga daqingensis CGMCC 1.7049T
Solimonas fluminis HRBBT
Sinimarinibacterium arenosum CAU 1509T
Sinimarinibacterium flocculans DSM 104150T
Fontimonas thermophila DSM 23609T
Solimonas aquatica DSM 25927T
Solimonas terrae KIS83-12T
Solimonas marina C16B3T
Solimonas soli DSM 21787T
Solimonas variicoloris DSM 15731T
Solimonas flava DSM 18980T
Ahniella affigens D13T
Tahibacter aquaticus DSM 21667T
Rudaea cellulosilytica DSM 22992T
Pseudolysobacter antarcticus AQ6296T
Dokdonella fugitiva A3T
Dokdonella koreensis DS123T
92
91
88

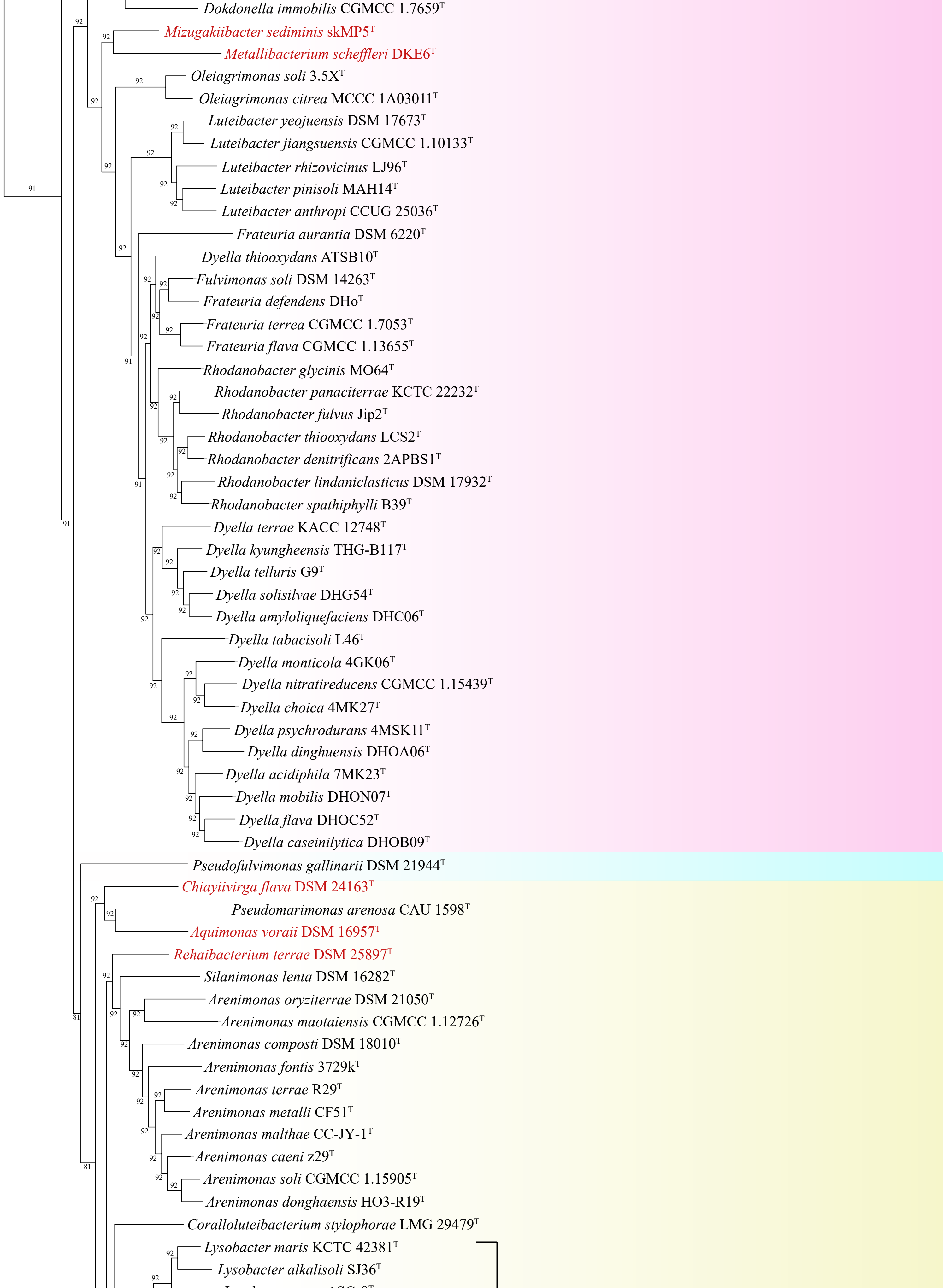
Dokdonella immobilis CGMCC 1.7659T
Mizugakiibacter sediminis skMP5T
Metallibacterium scheffleri DKE6T
Oleiagrimonas soli 3.5XT
Oleiagrimonas citrea MCCC 1A03011T
Luteibacter yeojuensis DSM 17673T
Luteibacter jiangsuensis CGMCC 1.10133T
Luteibacter rhizovicinus LJ96T
Luteibacter pinisoli MAH14T
Luteibacter anthropi CCUG 25036T
Frateuria aurantia DSM 6220T
Dyella thiooxydans ATSB10T
Fulvimonas soli DSM 14263T
Frateuria defendens DHoT
Frateuria terrea CGMCC 1.7053T
Frateuria flava CGMCC 1.13655T
Rhodanobacter glycinis MO64T
Rhodanobacter panaciterrae KCTC 22232T
Rhodanobacter fulvus Jip2T
Rhodanobacter thiooxydans LCS2T
Rhodanobacter denitrificans 2APBS1T
Rhodanobacter lindaniclasticus DSM 17932T
Rhodanobacter spathiphylli B39T
Dyella terrae KACC 12748T
Dyella kyungheensis THG-B117T
Dyella telluris G9T
Dyella solisilvae DHG54T
Dyella amyloliquefaciens DHC06T
Dyella tabacisoli L46T
Dyella monticola 4GK06T
Dyella nitratireducens CGMCC 1.15439T
Dyella choica 4MK27T
Dyella psychrodurans 4MSK11T
Dyella dinghuensis DHOA06T
Dyella acidiphila 7MK23T
Dyella mobilis DHON07T
Dyella flava DHOC52T
Dyella caseinilytica DHOB09T
Pseudofulvimonas gallinarii DSM 21944T
Chiayiivirga flava DSM 24163T
Pseudomarimonas arenosa CAU 1598T
Aquimonas voraii DSM 16957T
Rehaibacterium terrae DSM 25897T
Silanimonas lenta DSM 16282T
Arenimonas oryziterrae DSM 21050T
Arenimonas maotaiensis CGMCC 1.12726T
Arenimonas composti DSM 18010T
Arenimonas fontis 3729kT
Arenimonas terrae R29T
Arenimonas metalli CF51T
Arenimonas malthae CC-JY-1T
Arenimonas caeni z29T
Arenimonas soli CGMCC 1.15905T
Arenimonas donghaensis HO3-R19T
Coralloluteibacterium stylophorae LMG 29479T
Lysobacter maris KCTC 42381T
Lysobacter alkalisoli SJ36T

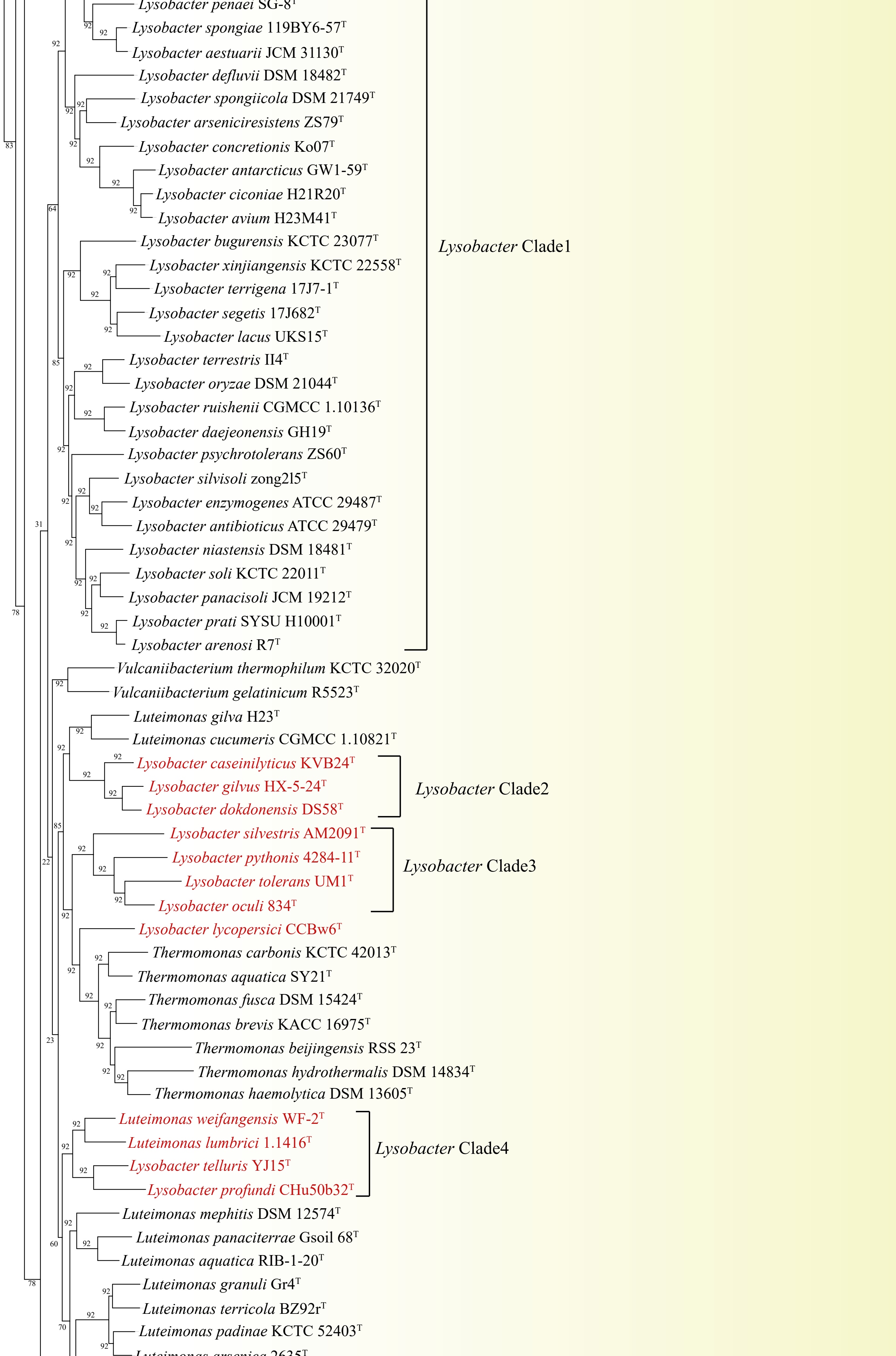
Lysobacter penaei SG-8T
Lysobacter spongiae 119BY6-57T
Lysobacter aestuarii JCM 31130T
Lysobacter defluvii DSM 18482T
Lysobacter spongiicola DSM 21749T
Lysobacter arseniciresistens ZS79T
Lysobacter concretionis Ko07T
Lysobacter antarcticus GW1-59T
Lysobacter ciconiae H21R20T
Lysobacter avium H23M41T
Lysobacter bugurensis KCTC 23077T
Lysobacter xinjiangensis KCTC 22558T
Lysobacter terrigena 17J7-1T
Lysobacter segetis 17J682T
Lysobacter lacus UKS15T
Lysobacter terrestris II4T
Lysobacter oryzae DSM 21044T
Lysobacter ruishenii CGMCC 1.10136T
Lysobacter daejeonensis GH19T
Lysobacter psychrotolerans ZS60T
Lysobacter silvisoli zong2l5T
Lysobacter enzymogenes ATCC 29487T
Lysobacter antibioticus ATCC 29479T
Lysobacter niastensis DSM 18481T
Lysobacter soli KCTC 22011T
Lysobacter panacisoli JCM 19212T
Lysobacter prati SYSU H10001T
Lysobacter arenosi R7T
Lysobacter Clade1
Vulcaniibacterium thermophilum KCTC 32020T
Vulcaniibacterium gelatinicum R5523T
Luteimonas gilva H23T
Luteimonas cucumeris CGMCC 1.10821T
Lysobacter caseinilyticus KVB24T
Lysobacter gilvus HX-5-24T
Lysobacter dokdonensis DS58T
Lysobacter Clade2
Lysobacter silvestris AM2091T
Lysobacter pythonis 4284-11T
Lysobacter tolerans UM1T
Lysobacter oculi 834T
Lysobacter Clade3
Lysobacter lycopersici CCBw6T
Thermomonas carbonis KCTC 42013T
Thermomonas aquatica SY21T
Thermomonas fusca DSM 15424T
Thermomonas brevis KACC 16975T
Thermomonas beijingensis RSS 23T
Thermomonas hydrothermalis DSM 14834T
Thermomonas haemolytica DSM 13605T
Luteimonas weifangensis WF-2T
Luteimonas lumbrici 1.1416T
Lysobacter telluris YJ15T
Lysobacter profundi CHu50b32T
Lysobacter Clade4
Luteimonas mephitis DSM 12574T
Luteimonas panaciterrae Gsoil 68T
Luteimonas aquatica RIB-1-20T
Luteimonas granuli Gr4T
Luteimonas terricola BZ92rT
Luteimonas padinae KCTC 52403T

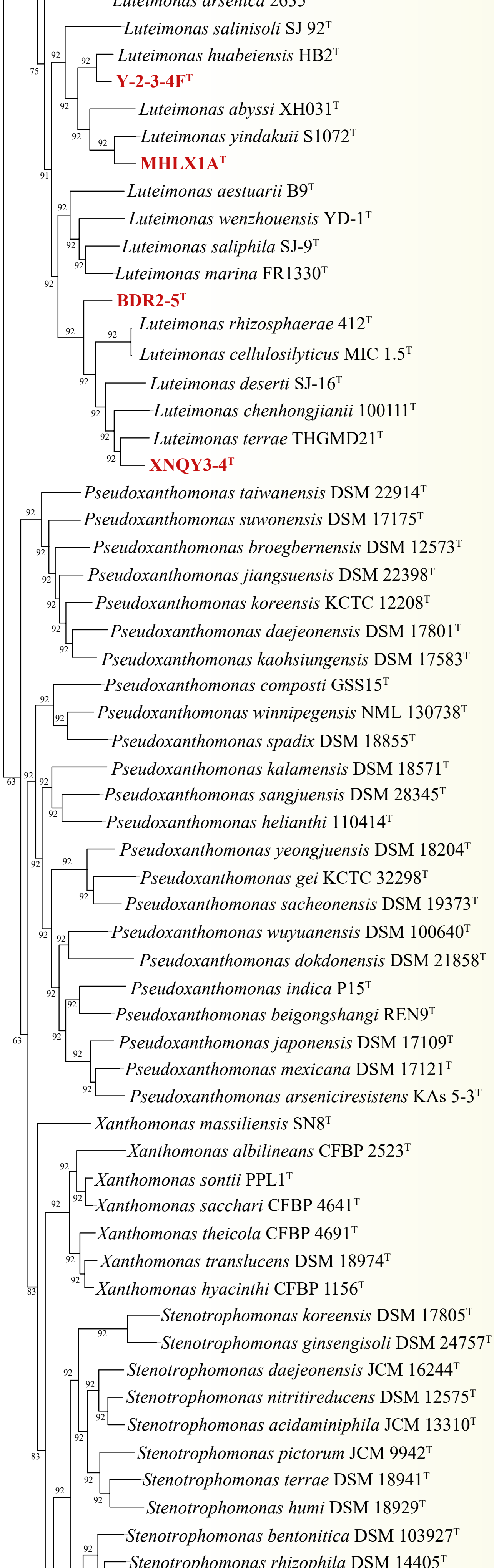
Luteimonas arsenica 2635
Luteimonas salinisoli SJ 92T
Luteimonas huabeiensis HB2T
Y-2-3-4FT
Luteimonas abyssi XH031T
Luteimonas yindakuii S1072T
MHLX1AT
Luteimonas aestuarii B9T
Luteimonas wenzhouensis YD-1T
Luteimonas saliphila SJ-9T
Luteimonas marina FR1330T
BDR2-5T
Luteimonas rhizosphaerae 412T
Luteimonas cellulosilyticus MIC 1.5T
Luteimonas deserti SJ-16T
Luteimonas chenhongjianii 100111T
Luteimonas terrae THGMD21T
XNQY3-4T
Pseudoxanthomonas taiwanensis DSM 22914T
Pseudoxanthomonas suwonensis DSM 17175T
Pseudoxanthomonas broegbernensis DSM 12573T
Pseudoxanthomonas jiangsuensis DSM 22398T
Pseudoxanthomonas koreensis KCTC 12208T
Pseudoxanthomonas daejeonensis DSM 17801T
Pseudoxanthomonas kaohsiungensis DSM 17583T
Pseudoxanthomonas composti GSS15T
Pseudoxanthomonas winnipegensis NML 130738T
Pseudoxanthomonas spadix DSM 18855T
Pseudoxanthomonas kalamensis DSM 18571T
Pseudoxanthomonas sangjuensis DSM 28345T
Pseudoxanthomonas helianthi 110414T
Pseudoxanthomonas yeongjuensis DSM 18204T
Pseudoxanthomonas gei KCTC 32298T
Pseudoxanthomonas sacheonensis DSM 19373T
Pseudoxanthomonas wuyuanensis DSM 100640T
Pseudoxanthomonas dokdonensis DSM 21858T
Pseudoxanthomonas indica P15T
Pseudoxanthomonas beigongshangi REN9T
Pseudoxanthomonas japonensis DSM 17109T
Pseudoxanthomonas mexicana DSM 17121T
Pseudoxanthomonas arseniciresistens KAs 5-3T
Xanthomonas massiliensis SN8T
Xanthomonas albilineans CFBP 2523T
Xanthomonas sontii PPL1T
Xanthomonas sacchari CFBP 4641T
Xanthomonas theicola CFBP 4691T
Xanthomonas translucens DSM 18974T
Xanthomonas hyacinthi CFBP 1156T
Stenotrophomonas koreensis DSM 17805T
Stenotrophomonas ginsengisoli DSM 24757T
Stenotrophomonas daejeonensis JCM 16244T
Stenotrophomonas nitritireducens DSM 12575T
Stenotrophomonas acidaminiphila JCM 13310T
Stenotrophomonas pictorum JCM 9942T
Stenotrophomonas terrae DSM 18941T
Stenotrophomonas humi DSM 18929T
Stenotrophomonas bentonitica DSM 103927T
Stenotrophomonas rhizophila DSM 14405T

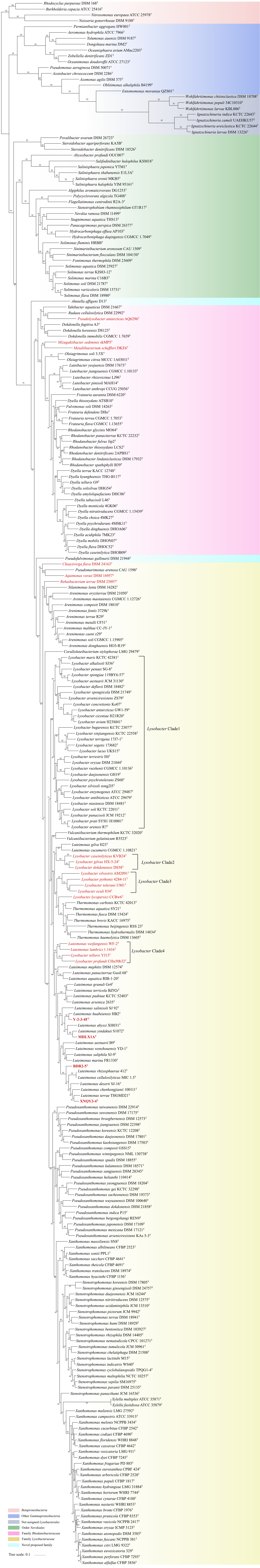

Figure S2. The UBCG phylogenetic tree among strains in order *Lysobacterales* based on 92 single copy core gene sequences. The strains in order *Nevskiales* and class *Betaproteobacteria* were used as outgroup. The scale bar corresponds to 0.1

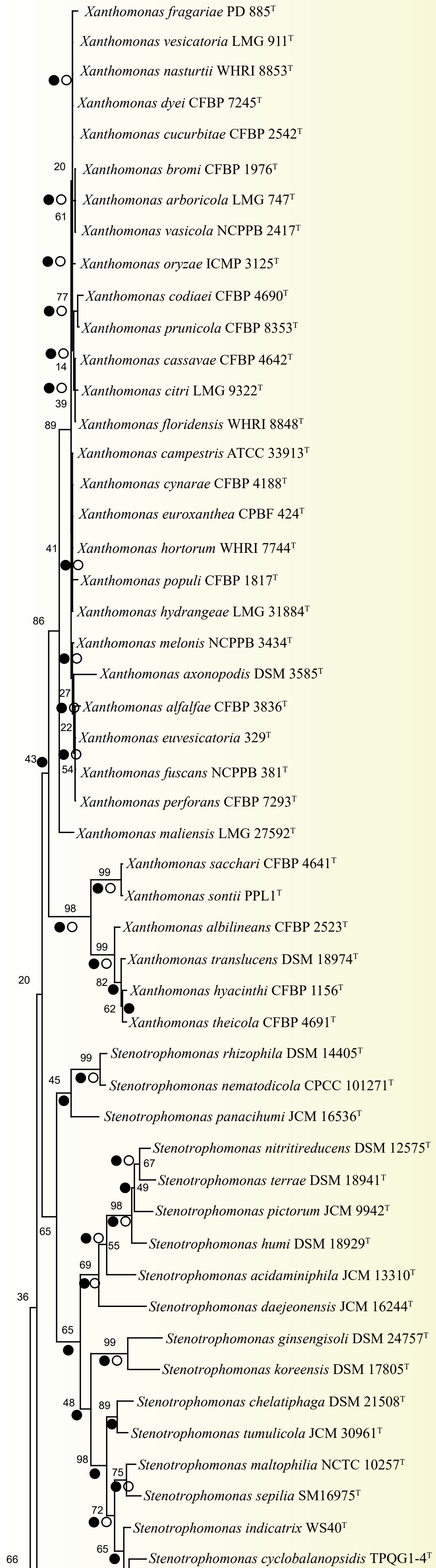

Xanthomonas fragariae PD 885^{T}
Xanthomonas vesicatoria LMG 911^{T}
Xanthomonas nasturtii WHRI 8853^{T}
Xanthomonas dyei CFBP 7245^{T}
Xanthomonas cucurbitae CFBP 2542^{T}
Xanthomonas bromi CFBP 1976^{T}
Xanthomonas arboricola LMG 747^{T}
Xanthomonas vasicola NCPPB 2417^{T}
Xanthomonas oryzae ICMP 3125^{T}
Xanthomonas codiaei CFBP 4690^{T}
Xanthomonas prunicola CFBP 8353^{T}
Xanthomonas cassavae CFBP 4642^{T}
Xanthomonas citri LMG 9322^{T}
Xanthomonas floridensis WHRI 8848^{T}
Xanthomonas campestris ATCC 33913^{T}
Xanthomonas cynarae CFBP 4188^{T}
Xanthomonas euroxanthea CPBF 424^{T}
Xanthomonas hortorum WHRI 7744^{T}
Xanthomonas populi CFBP 1817^{T}
Xanthomonas hydrangeae LMG 31884^{T}
Xanthomonas melonis NCPPB 3434^{T}
Xanthomonas axonopodis DSM 3585^{T}
Xanthomonas alfalfae CFBP 3836^{T}
Xanthomonas euvesicatoria 329^{T}
Xanthomonas fuscans NCPPB 381^{T}
Xanthomonas perforans CFBP 7293^{T}
Xanthomonas maliensis LMG 27592^{T}
Xanthomonas sacchari CFBP 4641^{T}
Xanthomonas sontii PPL1^{T}
Xanthomonas albilineans CFBP 2523^{T}
Xanthomonas translucens DSM 18974^{T}
Xanthomonas hyacinthi CFBP 1156^{T}
Xanthomonas theicola CFBP 4691^{T}
Stenotrophomonas rhizophila DSM 14405^{T}
Stenotrophomonas nematodicola CPCC 101271^{T}
Stenotrophomonas panacihumi JCM 16536^{T}
Stenotrophomonas nitritireducens DSM 12575^{T}
Stenotrophomonas terrae DSM 18941^{T}
Stenotrophomonas pictorum JCM 9942^{T}
Stenotrophomonas humi DSM 18929^{T}
Stenotrophomonas acidaminiphila JCM 13310^{T}
Stenotrophomonas daejeonensis JCM 16244^{T}
Stenotrophomonas ginsengisoli DSM 24757^{T}
Stenotrophomonas koreensis DSM 17805^{T}
Stenotrophomonas chelatiphaga DSM 21508^{T}
Stenotrophomonas tumulicola JCM 30961^{T}
Stenotrophomonas maltophilia NCTC 10257^{T}
Stenotrophomonas sepilia SM16975^{T}
Stenotrophomonas indicatrix WS40^{T}
Stenotrophomonas cyclobalanopsidis TPQG1-4^{T}

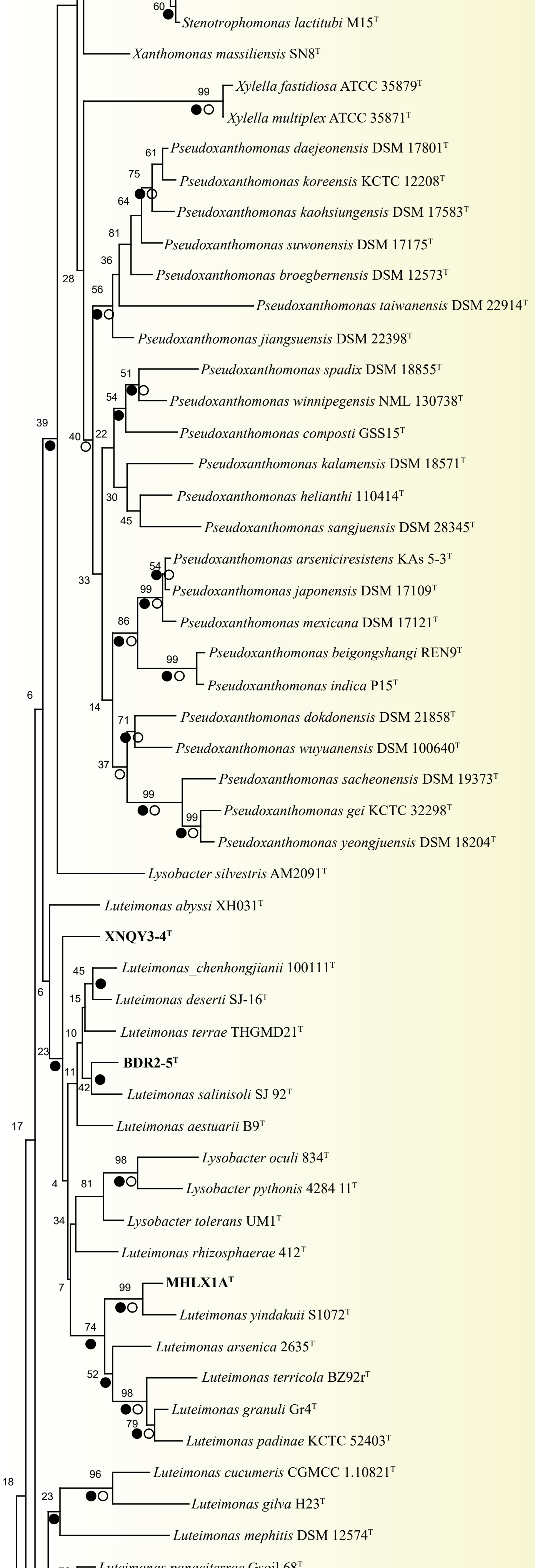

60
Stenotrophomonas lactitubi M15T
Xanthomonas massiliensis SN8T
99
Xylella fastidiosa ATCC 35879T
Xylella multiplex ATCC 35871T
61
Pseudoxanthomonas daejeonensis DSM 17801T
75
Pseudoxanthomonas koreensis KCTC 12208T
64
Pseudoxanthomonas kaohsiungensis DSM 17583T
81
Pseudoxanthomonas suwonensis DSM 17175T
36
Pseudoxanthomonas broegbernensis DSM 12573T
28
56
Pseudoxanthomonas taiwanensis DSM 22914T
Pseudoxanthomonas jiangsuensis DSM 22398T
51
Pseudoxanthomonas spadix DSM 18855T
54
Pseudoxanthomonas winnipegensis NML 130738T
39
40
22
Pseudoxanthomonas composti GSS15T
Pseudoxanthomonas kalamensis DSM 18571T
30
Pseudoxanthomonas helianthi 110414T
45
Pseudoxanthomonas sangjuensis DSM 28345T
Pseudoxanthomonas arseniciresistens KAs 5-3T
54
99
Pseudoxanthomonas japonensis DSM 17109T
33
86
Pseudoxanthomonas mexicana DSM 17121T
99
Pseudoxanthomonas beigongshangi REN9T
Pseudoxanthomonas indica P15T
6
14
71
Pseudoxanthomonas dokdonensis DSM 21858T
Pseudoxanthomonas wuyuanensis DSM 100640T
37
Pseudoxanthomonas sacheonensis DSM 19373T
99
99
Pseudoxanthomonas gei KCTC 32298T
Pseudoxanthomonas yeongjuensis DSM 18204T
Lysobacter silvestris AM2091T
Luteimonas abyssi XH031T
XNQY3-4T
45
Luteimonas_chenhongjianii 100111T
6
15
Luteimonas deserti SJ-16T
10
Luteimonas terrae THGMD21T
23
BDR2-5T
11
42
Luteimonas salinisoli SJ 92T
17
Luteimonas aestuarii B9T
98
Lysobacter oculi 834T
4
81
Lysobacter pythonis 4284 11T
34
Lysobacter tolerans UM1T
Luteimonas rhizosphaerae 412T
7
99
MHLX1AT
Luteimonas yindakuii S1072T
74
Luteimonas arsenica 2635T
52
Luteimonas terricola BZ92rT
98
Luteimonas granuli Gr4T
79
Luteimonas padinae KCTC 52403T
18
96
Luteimonas cucumeris CGMCC 1.10821T
23
Luteimonas gilva H23T
Luteimonas mephitis DSM 12574T
Luteimonas panaciterrae Gsoil 68T

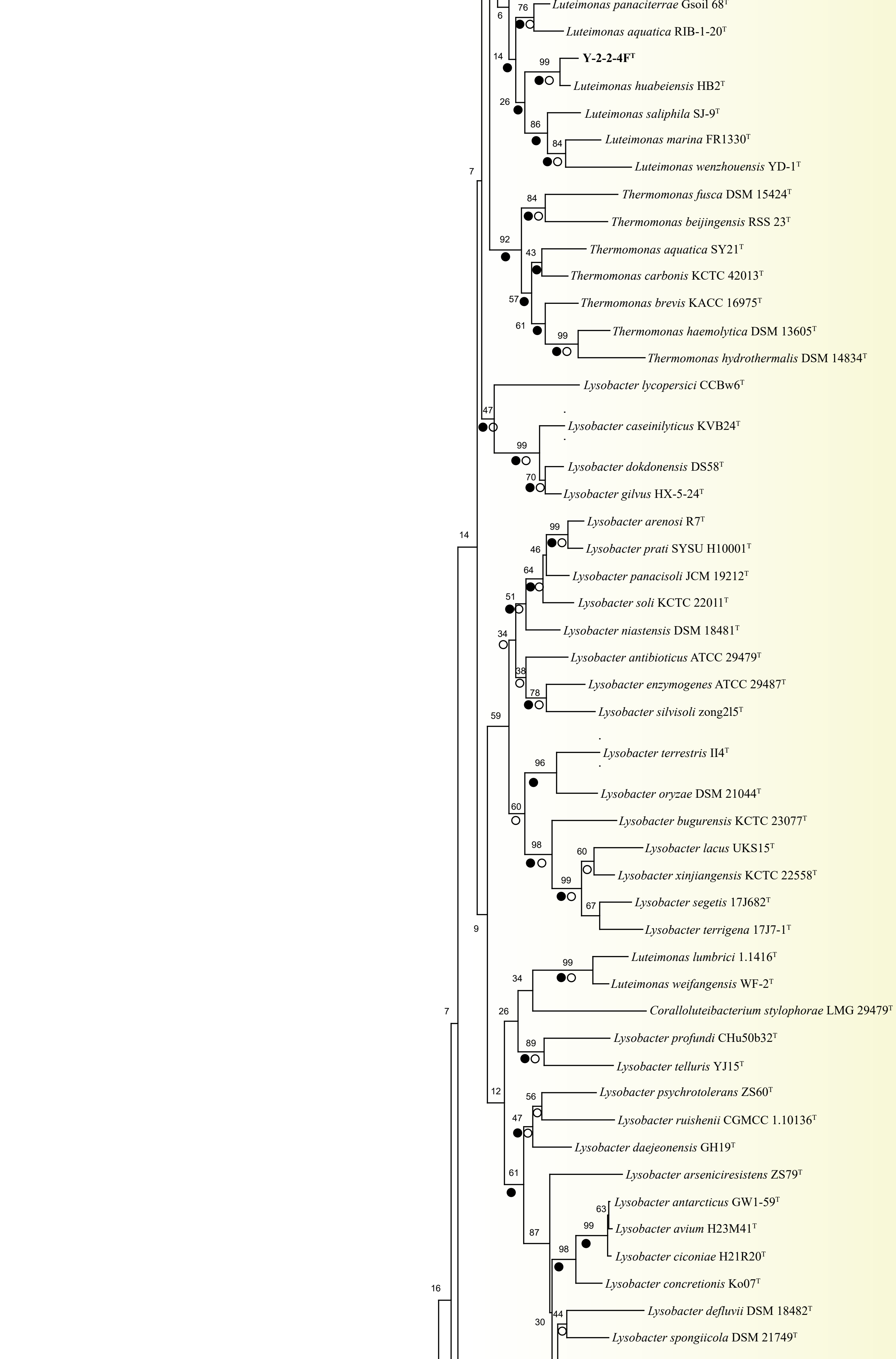
Luteimonas panaciterrae Gsoil 68T
Luteimonas aquatica RIB-1-20T
Y-2-2-4FT
Luteimonas huabeiensis HB2T
Luteimonas saliphila SJ-9T
Luteimonas marina FR1330T
Luteimonas wenzhouensis YD-1T
Thermomonas fusca DSM 15424T
Thermomonas beijingensis RSS 23T
Thermomonas aquatica SY21T
Thermomonas carbonis KCTC 42013T
Thermomonas brevis KACC 16975T
Thermomonas haemolytica DSM 13605T
Thermomonas hydrothermalis DSM 14834T
Lysobacter lycopersici CCBw6T
Lysobacter caseinilyticus KVB24T
Lysobacter dokdonensis DS58T
Lysobacter gilvus HX-5-24T
Lysobacter arenosi R7T
Lysobacter prati SYSU H10001T
Lysobacter panacisoli JCM 19212T
Lysobacter soli KCTC 22011T
Lysobacter niastensis DSM 18481T
Lysobacter antibioticus ATCC 29479T
Lysobacter enzymogenes ATCC 29487T
Lysobacter silvisoli zong2l5T
Lysobacter terrestris II4T
Lysobacter oryzae DSM 21044T
Lysobacter bugurensis KCTC 23077T
Lysobacter lacus UKS15T
Lysobacter xinjiangensis KCTC 22558T
Lysobacter segetis 17J682T
Lysobacter terrigena 17J7-1T
Luteimonas lumbrici 1.1416T
Luteimonas weifangensis WF-2T
Coralloluteibacterium stylophorae LMG 29479T
Lysobacter profundi CHu50b32T
Lysobacter telluris YJ15T
Lysobacter psychrotolerans ZS60T
Lysobacter ruishenii CGMCC 1.10136T
Lysobacter daejeonensis GH19T
Lysobacter arseniciresistens ZS79T
Lysobacter antarcticus GW1-59T
Lysobacter avium H23M41T
Lysobacter ciconiae H21R20T
Lysobacter concretionis Ko07T
Lysobacter defluvii DSM 18482T
Lysobacter spongiicola DSM 21749T

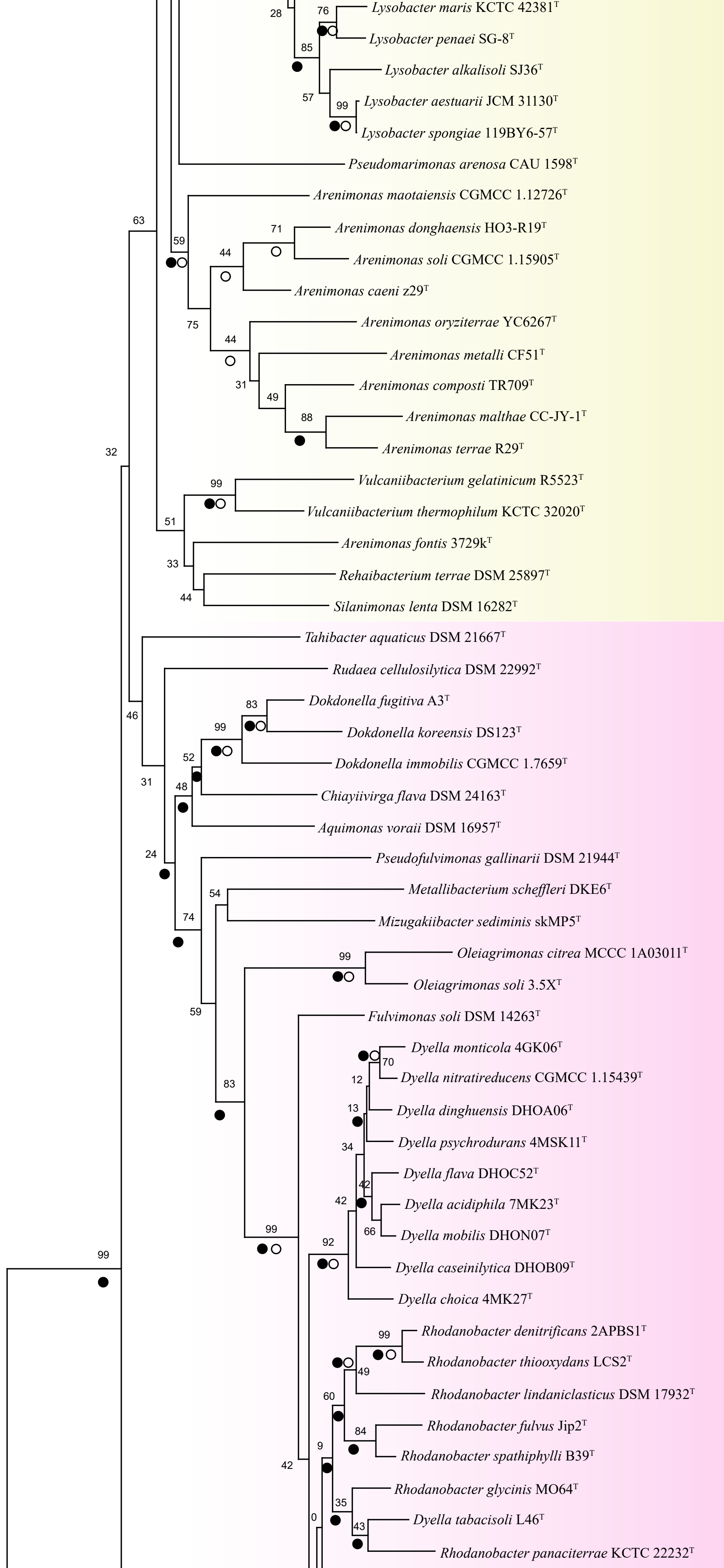
Lysobacter maris KCTC 42381T
Lysobacter penaei SG-8T
Lysobacter alkalisoli SJ36T
Lysobacter aestuarii JCM 31130T
Lysobacter spongiae 119BY6-57T
Pseudomarimonas arenosa CAU 1598T
Arenimonas maotaiensis CGMCC 1.12726T
Arenimonas donghaensis HO3-R19T
Arenimonas soli CGMCC 1.15905T
Arenimonas caeni z29T
Arenimonas oryziterrae YC6267T
Arenimonas metalli CF51T
Arenimonas composti TR709T
Arenimonas malthae CC-JY-1T
Arenimonas terrae R29T
Vulcaniibacterium gelatinicum R5523T
Vulcaniibacterium thermophilum KCTC 32020T
Arenimonas fontis 3729kT
Rehaibacterium terrae DSM 25897T
Silanimonas lenta DSM 16282T
Tahibacter aquaticus DSM 21667T
Rudaea cellulosilytica DSM 22992T
Dokdonella fugitiva A3T
Dokdonella koreensis DS123T
Dokdonella immobilis CGMCC 1.7659T
Chiayiivirga flava DSM 24163T
Aquimonas voraii DSM 16957T
Pseudofulvimonas gallinarii DSM 21944T
Metallibacterium scheffleri DKE6T
Mizugakiibacter sediminis skMP5T
Oleiagrimonas citrea MCCC 1A03011T
Oleiagrimonas soli 3.5XT
Fulvimonas soli DSM 14263T
Dyella monticola 4GK06T
Dyella nitratireducens CGMCC 1.15439T
Dyella dinghuensis DHOA06T
Dyella psychrodurans 4MSK11T
Dyella flava DHOC52T
Dyella acidiphila 7MK23T
Dyella mobilis DHON07T
Dyella caseinilytica DHOB09T
Dyella choica 4MK27T
Rhodanobacter denitrificans 2APBS1T
Rhodanobacter thiooxydans LCS2T
Rhodanobacter lindaniclasticus DSM 17932T
Rhodanobacter fulvus Jip2T
Rhodanobacter spathiphylli B39T
Rhodanobacter glycinis MO64T
Dyella tabacisoli L46T
Rhodanobacter panaciterrae KCTC 22232T

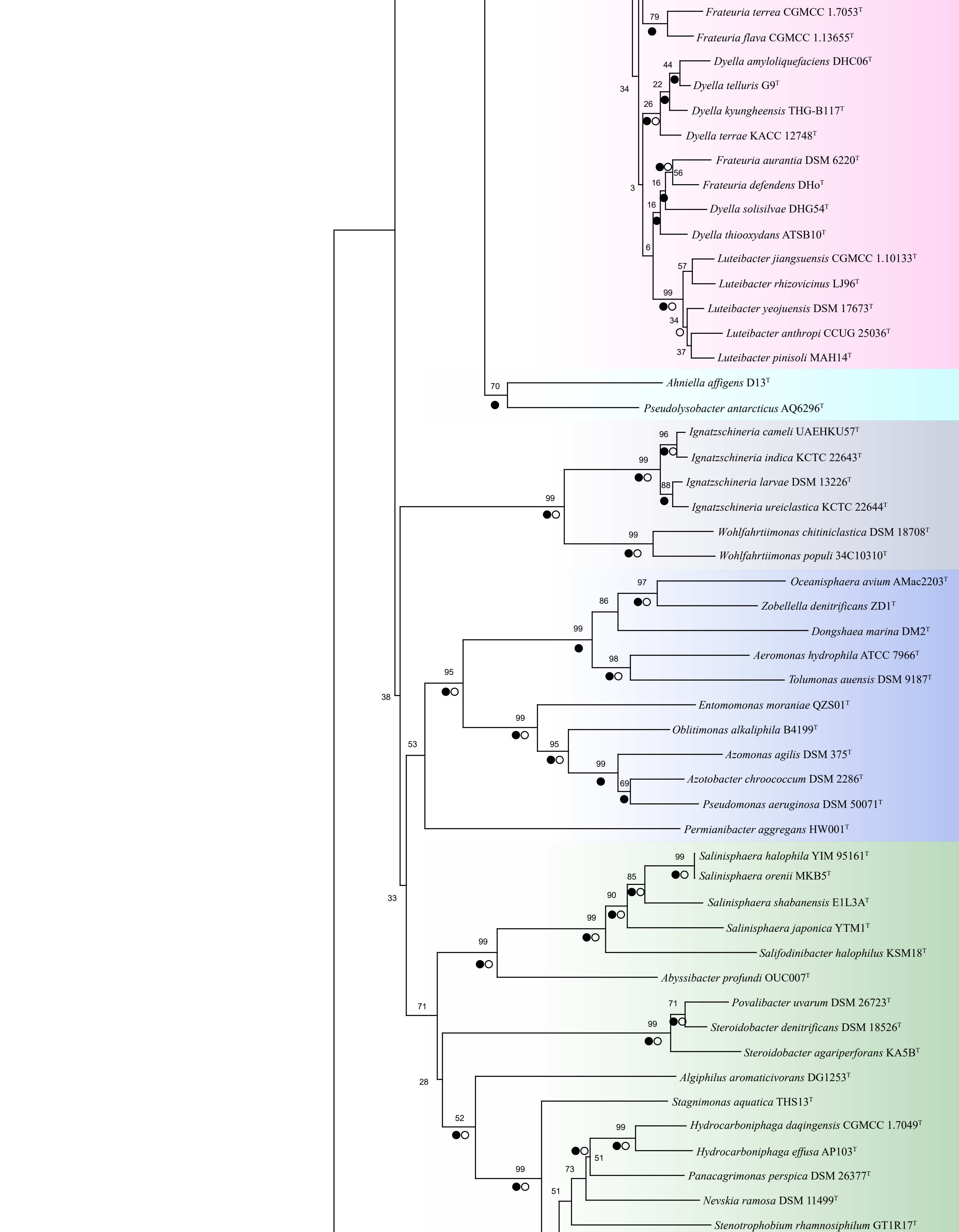

Frateuria terrea CGMCC 1.7053T
Frateuria flava CGMCC 1.13655T
Dyella amyloliquefaciens DHC06T
Dyella telluris G9T
Dyella kyungheensis THG-B117T
Dyella terrae KACC 12748T
Frateuria aurantia DSM 6220T
Frateuria defendens DHoT
Dyella solisilvae DHG54T
Dyella thiooxydans ATSB10T
Luteibacter jiangsuensis CGMCC 1.10133T
Luteibacter rhizovicinus LJ96T
Luteibacter yeojuensis DSM 17673T
Luteibacter anthropi CCUG 25036T
Luteibacter pinisoli MAH14T
Ahniella affigens D13T
Pseudolysobacter antarcticus AQ6296T
Ignatzschineria cameli UAEHKU57T
Ignatzschineria indica KCTC 22643T
Ignatzschineria larvae DSM 13226T
Ignatzschineria ureiclastica KCTC 22644T
Wohlfahrtiimonas chitiniclastica DSM 18708T
Wohlfahrtiimonas populi 34C10310T
Oceanisphaera avium AMac2203T
Zobellella denitrificans ZD1T
Dongshaea marina DM2T
Aeromonas hydrophila ATCC 7966T
Tolumonas auensis DSM 9187T
Entomomonas moraniae QZS01T
Oblitimonas alkaliphila B4199T
Azomonas agilis DSM 375T
Azotobacter chroococcum DSM 2286T
Pseudomonas aeruginosa DSM 50071T
Permianibacter aggregans HW001T
Salinisphaera halophila YIM 95161T
Salinisphaera orenii MKB5T
Salinisphaera shabanensis E1L3AT
Salinisphaera japonica YTM1T
Salifodinibacter halophilus KSM18T
Abyssibacter profundi OUC007T
Povalibacter uvarum DSM 26723T
Steroidobacter denitrificans DSM 18526T
Steroidobacter agariperforans KA5BT
Algiphilus aromaticivorans DG1253T
Stagnimonas aquatica THS13T
Hydrocarboniphaga daqingensis CGMCC 1.7049T
Hydrocarboniphaga effusa AP103T
Panacagrimonas perspica DSM 26377T
Nevskia ramosa DSM 11499T
Stenotrophobium rhamnosiphilum GT1R17T

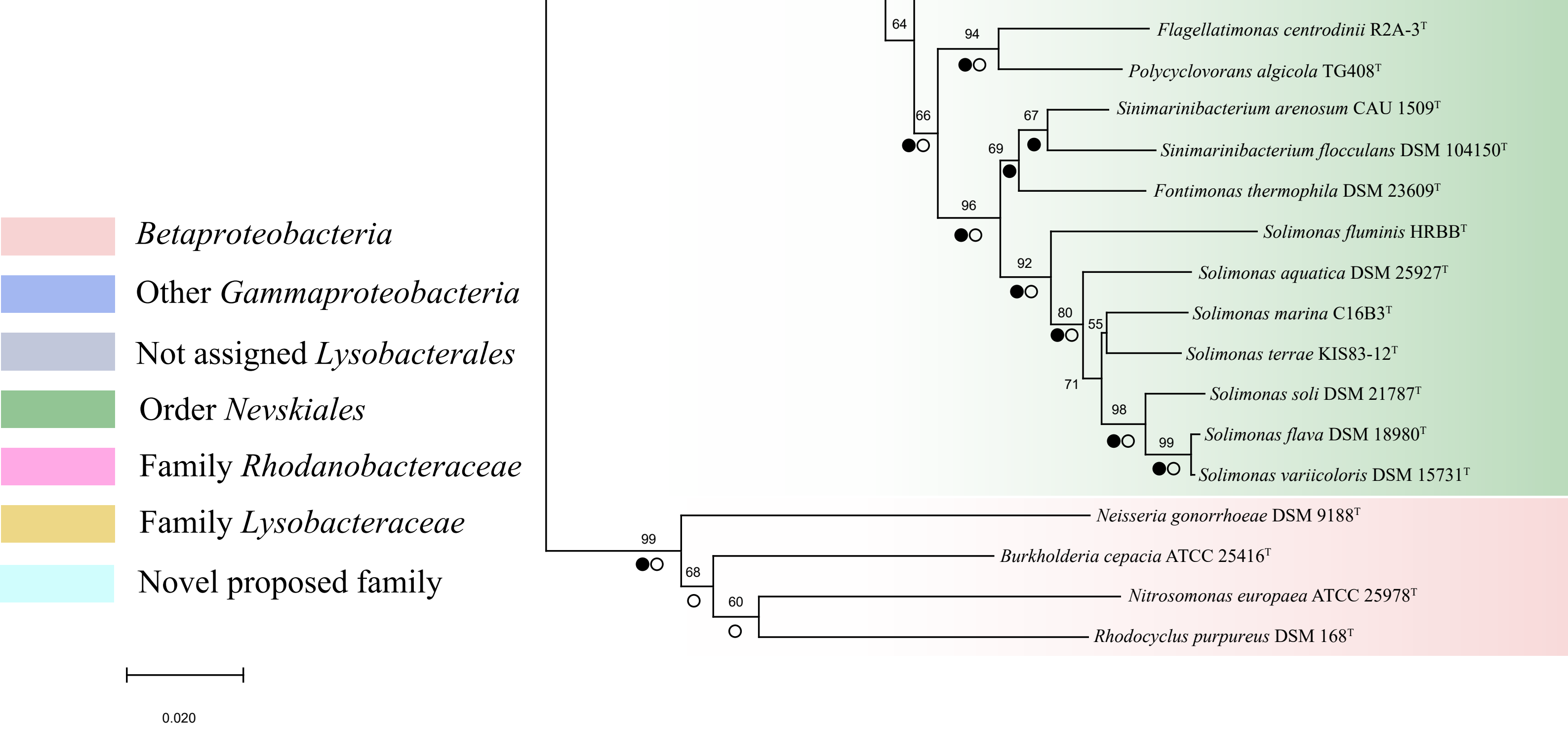

Figure S3. Neighbor-joining tree showing phylogenetic relationships among the strains in order *Lysobacterales* based on 16S rRNA gene sequences. The strains in order *Nevskiales* and class *Betaproteobacteria* were used as outgroup. The scale bar corresponds to 0.01 substitutions per nucleotide site. Filled circles indicate branches recovered by maximum-likelihood method and open circles at branches recovered by the maximumparsimony method.

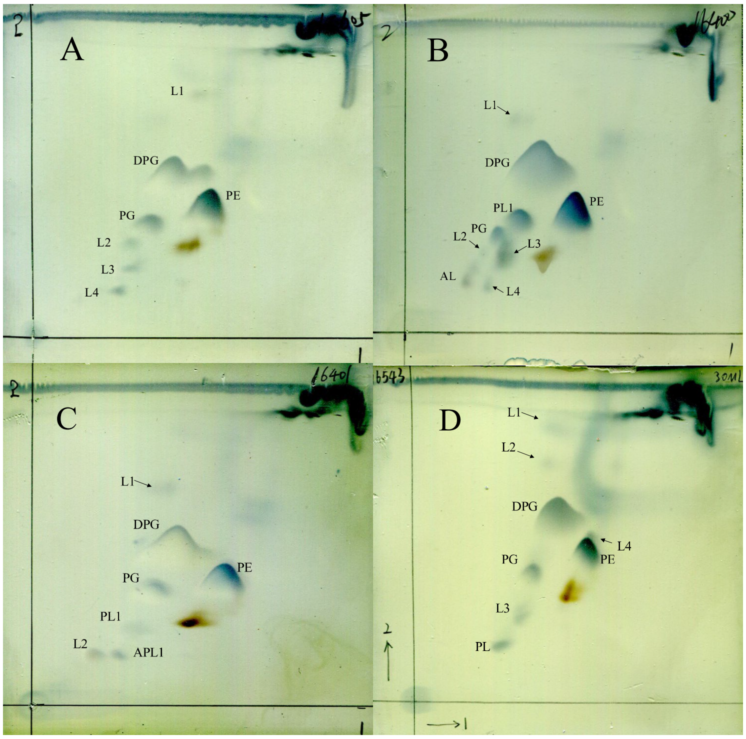

Figure S4 . Polar lipid profiles of strain Y-2-2-4FT (A), MHLX1A (B), BDR2-5 (C) and XNQY3-4 (D) separated and detected by two-dimensional thin-layer chromatography and spraying with a molybdato-phosphoric acid reagent, respectively. DPG, disphosphatidylglycerol; PE, phosphatidylethanolamine; PG, phosphatidylglycerol; PL, phospholipid; AL, aminolipid; APL, unidentified aminophospholipid; L, unidentified lipid.

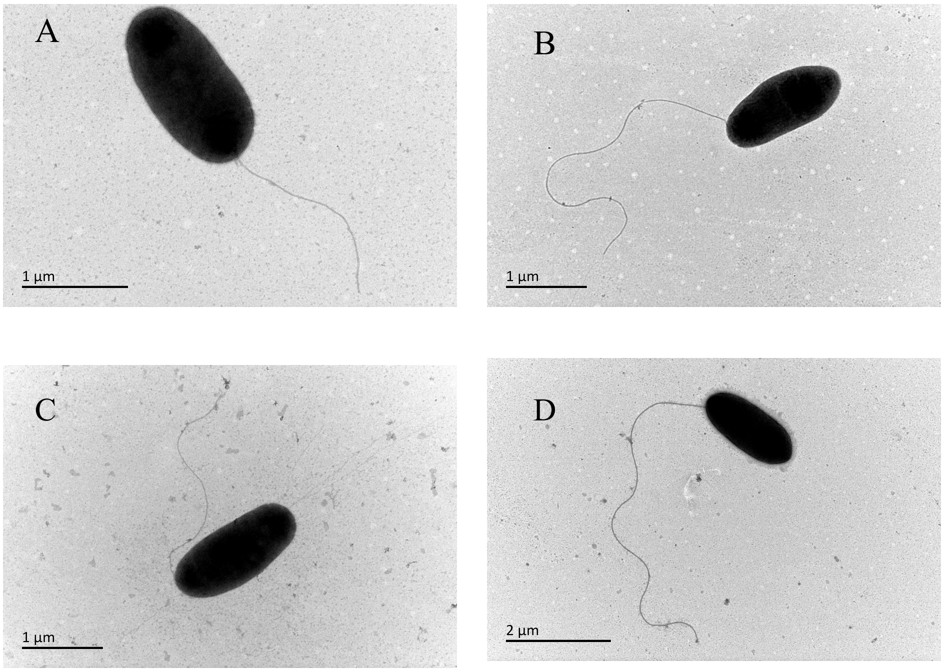

Figure S5 . Electron microscopy images of strain Y-2-2-4F (A), MHLX1A (B), BDR2-5 (C) and XNQY3-4 (D)

Table.S2 Cellular fatty acid profiles (%) of the four strains and closely related type strains.

Strains: 1, Y-2-2-4F^T; 2, MHLX1A^T; 3, BDR2-5^T; 4, XNQY3-4^T; 5, *Luteimonas huabeiensis* HB2^T; 6, *Luteimonas yindakuii* S-1072^T; 7, *Luteimonas chenhongjianii* 100111^T; 8, *Luteimonas terrae* THG-MD21^T; 9, *Luteimonas aestuarii* B9^T. NA, not available; -, not detected. *Summed features are the groups of fatty acids that cannot be distinguished by the MIDI system. Summed features 3 comprises $C_{16:1}\omega 7c$ and/or $C_{16:1}\omega 6c$. Summed features 9 comprises 10-methyl $C_{16:0}$ and/or iso-$C_{17:1}\omega 9c$.

Fatty acid	1	2	3	4	5	6	7	8	9
$C_{14:0}$	0.7	0.6	0.6	0.3	1.4	0.5	0.7	1.1	1.1
$C_{16:0}$	3.8	3.6	2.3	2.0	2.7	2.6	2.6	9.6	2.5
iso-$C_{10:0}$	-	0.1	-	0.2	0.5	4.9	21.5	1	0.5
iso-$C_{11:0}$	5.9	3.9	5.4	5.7	12.4	NA	NA	11.7	15.6
iso-$C_{14:0}$	1.6	0.4	-	0.7	4.9	1	NA	1.6	2.6
iso-$C_{15:0}$	25.8	28.4	31.6	18.9	39	15.5	18.1	14.2	18.5
anteiso-$C_{15:0}$	2.7	1.6	4.3	3.6	0.8	1.7	0.8	1.2	2
iso-$C_{16:0}$	16.2	5.0	0.7	9.8	8.5	20.8	2.2	9.9	11.9
iso-$C_{17:0}$	6.4	11.8	14.9	13.2	2.8	7.2	6.6	4.1	3.9
iso-$C_{11:0}$ 3-OH	1.8	2.6	2.9	2.7	3.7	2.4	5.9	4.1	8.5
iso-$C_{13:0}$ 3-OH	0.2	0.7	1.0	0.7	3	2.8	5.5	2.5	-
iso-$C_{17:0}\,\omega 7c$	-	-	-	-	-	-	21.1	14.4	-
Summed Features *	8.6	6.5	2.6	3.5	3.2	8.4	5.2	4.9	1.3
Summed features 3	19.4	30.7	25.5	33.2	9.5	26.2	-	-	25.9
Summed features 9	0.7	0.6	0.6	0.3	1.4	0.5	0.7	1.1	1.1

Data in column 5 is from [48], data in column 6 is from [49], data in column 7 is from [50], data in column 8 is from [51] and data in column 9 is from [52].
